# Identification of Candidate Ergosterol-Responsive Proteins Associated with the Plasma Membrane of *Arabidopsis thaliana*

**DOI:** 10.3390/ijms20061302

**Published:** 2019-03-14

**Authors:** Thembisile G. Khoza, Ian A. Dubery, Lizelle A. Piater

**Affiliations:** Department of Biochemistry, University of Johannesburg, Auckland Park, Johannesburg 2006, South Africa; tkhoza03@gmail.com (T.K.); idubery@uj.ac.za (I.D.)

**Keywords:** affinity chromatography, ergosterol, fungal perception, innate immunity, pattern recognition receptors, plasma membrane, proteomics

## Abstract

The impact of fungal diseases on crop production negatively reflects on sustainable food production and overall economic health. Ergosterol is the major sterol component in fungal membranes and regarded as a general elicitor or microbe-associated molecular pattern (MAMP) molecule. Although plant responses to ergosterol have been reported, the perception mechanism is still unknown. Here, *Arabidopsis thaliana* protein fractions were used to identify those differentially regulated following ergosterol treatment; additionally, they were subjected to affinity-based chromatography enrichment strategies to capture and categorize ergosterol-interacting candidate proteins using liquid chromatography coupled with tandem mass spectrometry (LC-MS/MS). Mature plants were treated with 250 nM ergosterol over a 24 h period, and plasma membrane-associated fractions were isolated. In addition, ergosterol was immobilized on two different affinity-based systems to capture interacting proteins/complexes. This resulted in the identification of defense-related proteins such as chitin elicitor receptor kinase (CERK), non-race specific disease resistance/harpin-induced (NDR1/HIN1)-like protein, Ras-related proteins, aquaporins, remorin protein, leucine-rich repeat (LRR)- receptor like kinases (RLKs), G-type lectin S-receptor-like serine/threonine-protein kinase (GsSRK), and glycosylphosphatidylinositol (GPI)-anchored protein. Furthermore, the results elucidated unknown signaling responses to this MAMP, including endocytosis, and other similarities to those previously reported for bacterial flagellin, lipopolysaccharides, and fungal chitin.

## 1. Introduction

Plants lack an adaptive immune system and solely depend on a multi-complex innate immunity to defend themselves. The first line of defense occurs on the plant cell surface, where membrane-bound pattern recognition receptors (PRRs) recognize conserved motifs within microbes. These microbe-associated molecular patterns (MAMPs) are typically essential components for microorganism functioning and include the bacterial flagellin epitope, flg22. This MAMP is recognized by the PRR receptor, flagellin sensitive 2 (FLS2), which was proven by showing that mutated epitope residues did not lead to flagellin perception but instead, susceptibility and infection was observed [1,2]. Similarly, a lipopolysaccharide (LPS) receptor was identified in the Brassicaceae family. It was found that *Arabidopsis thaliana* detected LPS of *Xanthomonas campestris* and *Pseudomonas* species using a bulb-type (B-type) lectin S-domain (SD)-1 receptor like kinase (RLK) termed lipooligosaccharide-specific reduced elicitation (LORE) [3]. The recognition of MAMPs by PRRs leads to activation of the primary defense termed microbe-triggered immunity (MTI). Due to the co-evolution of both microbes and host, several organisms have the ability to suppress MTI components by releasing virulent molecules called effectors, which leads to effector-triggered susceptibility (ETS). This marks the second line of defense, known as effector-triggered immunity (ETI), where these effectors are recognized by intracellular nucleotide-binding leucine-rich repeat (NB-LRR) proteins [4,5,6]. Subsequent processes include the transcription of defense genes and expression of pathogenesis-related (PR) proteins. General cellular events associated with MTI and ETI include changes in cytoplasmic Ca^2+^ levels, activation of mitogen-activated protein kinase (MAPK) cascades, bursts of reactive oxygen species (ROS) and nitric oxide (NO), deposition of callose to reinforce the cell wall, production of anti-microbial compounds such as phytoalexins, and often, localized cell death [4,7,8,9,10].

Currently, crop yield and food security are global concerns due to often devastating fungal–plant interactions [11], which also impact economies, particularly those of third world countries. Fungal MAMP molecules such as chitin and β-glucan have been shown to possess a common elicitor activity in various hosts irrespective of the different molecular structures. Here, the MAMP specific to this investigation is ergosterol, which is the major sterol component of the phospholipid bilayer of fungal cell membranes and functions in membrane stability and signaling. Ergosterol is found in several pathogens such as *Cladosporium fulvum* and *Botrytis cinerea,* but surprisingly some biotrophic fungi, including the powdery mildew (*Erysiphe cichoracearum)* and rust (*Puccinia triticina*) fungi, lack ergosterol [12]. Ergosterol contains two additional double bonds when compared to cholesterol and β-sitosterol, the most abundant phytosterol that is also an analogue of cholesterol [11,13]. Even with the aforementioned similarities of ergosterol to sitosterol, it is still perceived as a “non-self” MAMP [14], as has previously been shown in plant studies. Intracellular defense occurs within minutes in response to sub-nanomolar concentrations of ergosterol in tobacco and tomato cells. Included here is an increase of cytosolic Ca^2+^ levels, production of ROS, ion fluxes across the plasma membrane, protein phosphorylation, and production of phytoalexins [15,16,17,18,19,20,21,22]. It has been found that inhibiting the ergosterol biosynthesis pathway in colonizing fungi not only reduces fungal growth but also alters the sterol composition [12]. According to Dohnal et al. [23], ergosterol can be used as a fungal marker to evaluate infection levels in barley and corn crops, while treatment was also found to increase the expression of genes for PR1a, PR1b, PR3Q, and PR5 [16], acidic PR proteins used as markers for systemic acquired resistance (SAR) in host plants. Additionally, ergosterol elicitation has also shown expression of proteinase inhibitors, phenylalanine-ammonia lyase and sesquiterpene cyclase [16]. Although the perception mechanism is unknown, it is hypothesized that plants may possess an ergosterol receptor/receptor complex, or ergosterol penetrates the lipid bilayer and leads to perturbations of the plant cell system due to its ability to form stable microdomains in the plasma membrane [24,25]. In this study, we describe the use of proteomic approaches to identify differentially regulated plasma membrane-associated proteins following ergosterol treatment, as well as subsequent affinity-based chromatographic strategies of the said fraction to capture and enrich ergosterol-interacting candidate proteins so as to shed light on the unknown perception mechanism(s).

## 2. Results

### 2.1. Plasma Membrane (PM)-Associated Fraction Isolation and Verification

The plasma membrane (PM) outlines the interface between the cell and extracellular environment and is also the primary unit for signal recognition and transduction. Thus, elucidating and characterizing changes in the PM-associated proteome could identify possible receptor(s) and interacting/complementary complexes that are involved in immune responses to ergosterol. A challenge faced when extracting the PM proteome is the highly hydrophobic integral proteins that have a tendency of precipitating out of solution [26]. The conventional method of isolating PM proteins is the two-phase partitioning system, which requires 100–150 g of plant material [26]. However, the small-scale procedure has been found to result in PM-associated proteins comparable to the conventional method while employing much less starting material [26] and was the method followed in this investigation. The successful isolation of the PM-associated fraction during the ergosterol-treatment time course was routinely verified using Western Blot analysis (Figure S1) and the H^+^-ATPase assay. Furthermore, any non-PM-associated proteins were eliminated in the sequencing data analysis, as well as non-specific interacting proteins by the inclusion of control samples where no ergosterol was immobilized to the capture resins. Figure S2 shows the different isolated fractions with differentially regulated band intensities for each lane, thus implying successful enrichment of the PM-associated fraction. 

### 2.2. PM-Associated Ergosterol-Responsive Candidate Protein Identification

**Figure 1:**
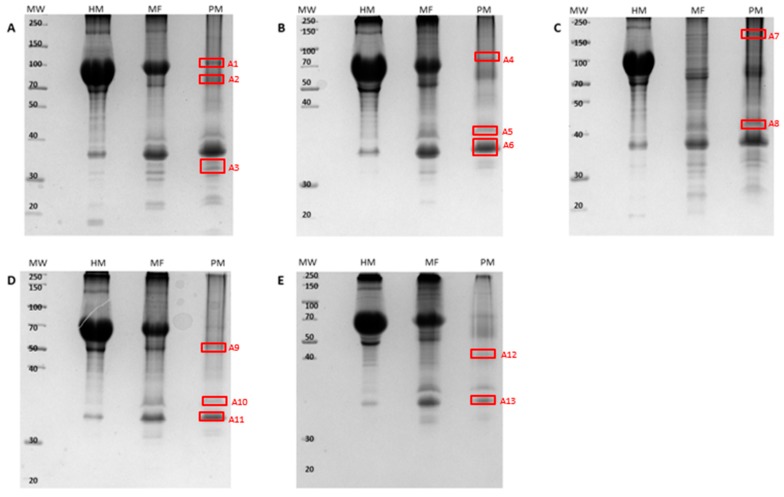
Representative 12% 1D-SDS PAGE gels stained with the Fairbanks method and showing the homogenate (HM), microsomal (MF), and plasma membrane (PM)-associated fractions subsequent to isolation. Gels represent all time point treatments with ergosterol, where **A** = control, **B** = 0 h treated, **C** = 6 h treated, **D** = 12 h treated, and **E** = 24 h treated. Equal volumes (20 µL) of the samples were mixed with 2X sample buffer, and electrophoresis was carried out at 90 V for 3 h. The red blocks indicate bands that were excised (A1–A13) for liquid chromatography coupled with tandem mass spectrometry (LC-MS/MS) identification.

**Figure 2:**
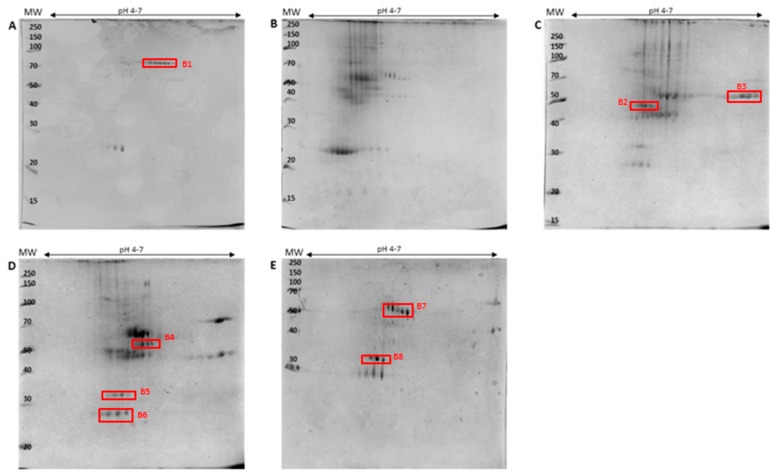
Comparative 2D-SDS-PAGE analysis for ergosterol-treated *Arabidopsis thaliana* PM-associated extracts. Proteins were precipitated with acetone, and 100 µg total protein was loaded onto immobilized pH gradient (IPG) strips, pH 4–7, for isoelectric focusing (IEF). The protein regulation differences are shown for **A** = control, and **B** = 0 h -, **C** = 6 h -, **D** = 12 h -, and **E** = 24 h-treated samples. The red blocks (B1–B8) indicate the protein spots excised for LC-MS/MS identification.

Data analysis was initially conducted on the ergosterol-induced PM-associated fractions subsequent to isolation and prior to enrichment. The results are shown for the 1D and 2D SDS-PAGE gels (Figure 1 and Figure 2) where differentially (densitometrically/electrophoretically) regulated bands/spots were selected for identification.

As previously mentioned, one band on a 1D gel may consist of multiple proteins. This emphasizes the need to identify the proteins affected/induced by ergosterol treatment and the role in perception of/response to this MAMP. Selected bands/spots from both the 1D- (Figure 1, A1–A13) and 2D SDS-PAGE (Figure 2, B1–B8) gels subsequent to ergosterol treatment were excised and prepared for liquid chromatography coupled with tandem mass spectrometry (LC-MS/MS) identification. The LC-MS/MS sequencing runs were repeated (separate experiments) for confirmation of protein lists obtained. The resulting spectra of the peptides were analyzed using the Byonic™ software (Protein Metrics, Cupertino, CA, USA). The program produces two plots, a protein score plot and mass error loadings plot (Figures S3 and S4). The protein score plot was used for the selection of proteins showing differential abundance or variable selection. This is known as the variable importance in projection (VIP) method and ranks proteins based on their contribution to the total variation of the samples. Differentially abundant proteins/peptides were selected on the VIP score where the set threshold was equal to one [27], and this value was presented as the log probability in all tables. The latter (as well as the Byonic score) determined the significance of the identified proteins. Even though these two said parameters could have been used individually, the values would have been less dependable. However, used together, they increased the significance. The dataset acquired was then normalized to the peptides of *Arabidopsis* proteins using the UniprotKB database. The identified *A. thaliana* PM-associated responsive proteins are summarized according to functional categories in Table 1 for the 1D SDS-PAGE bands and Table 2 for the 2D SDS-PAGE spots, respectively. There was better qualitative resolution for protein identification from the former to the latter. Furthermore, the differences between the theoretical and the experimental molecular weights (MW) for all proteins (low and high abundant) could be justified by the existence of structured water layers on the protein surface that affected the experimental MW determination on the SDS- PAGE [28].

### 2.3. Identification of PM-Associated Ergosterol-Interacting Candidate Proteins

#### 2.3.1. Epoxide Magnetic Microspheres-Based Ergosterol Immobilization

**Figure 3:**
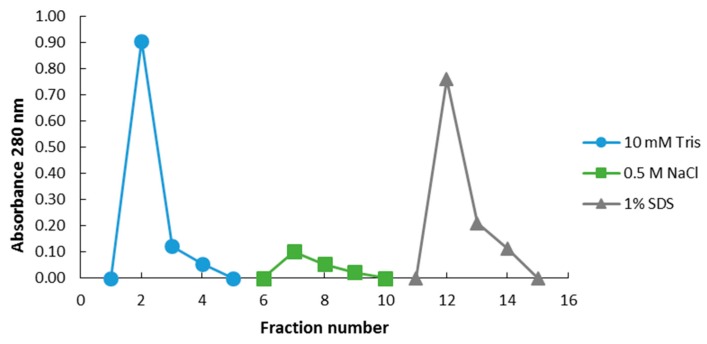
Representative elution profile of binding events between ergosterol-immobilized MagResyn™ magnetic microspheres and *A. thaliana* PM-associated proteins at 6 h following treatment. The blue curve represents the absorbance of the flow-through (unbound) fractions eluted with 10 mM Tris-HCl, pH 7.5. The green curve is the absorbance of the weakly bound proteins removed with 0.5 M NaCl, and the grey curve represents absorbance of proteins desorbed from the column with 1% SDS solution.

**Figure 4:**
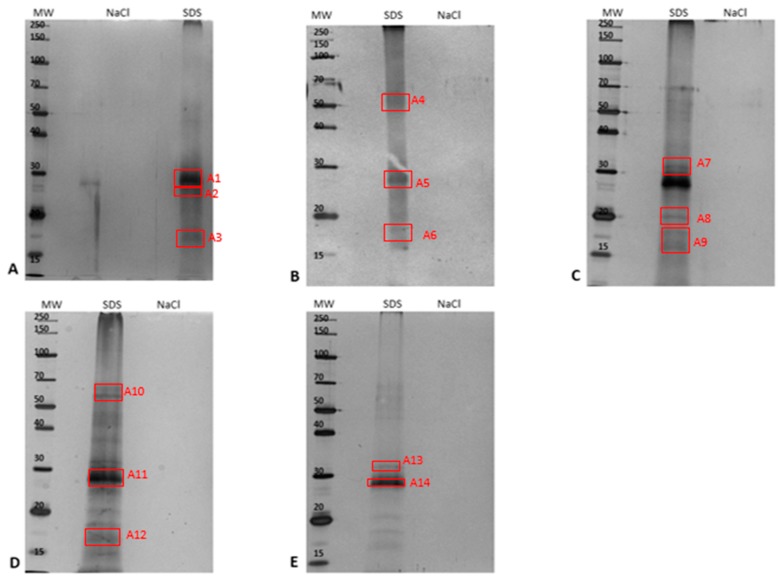
Comparative 12% 1D-SDS-PAGE analysis of ergosterol-interacting candidate proteins eluted with 0.5 NaCl and 1% SDS during the affinity-capture procedure using epoxide magnetic microspheres, where **A** = control, and **B** = 0 h-, **C** = 6 h-, **D** = 12 h-, and **E** = 24 h-treated samples. For each fraction, 20 µg total protein was loaded and electrophoresed at constant 90 V at room temperature. The red blocks (A1–A14) were excised subsequent to silver staining and analyzed using LC-MS/MS.

In order to capture and enrich ergosterol-interacting candidate proteins from the PM-associated leaf tissue fraction, MagResyn^TM^ magnetic microspheres were used. The binding and elution events that showed the resulting protein elution to changing in eluents is represented in Figure 3 for the PM-associated proteins following a 6 h treatment. The elution profiles for the other time points are presented in the Supplementary Data as Figures S5–S9. The NaCl and SDS fractions for each time study were analyzed by SDS-PAGE and are illustrated as Figure 4. Proteins eluted with 0.5 M NaCl were not detectable in contrast to those eluted with 1% SDS, which disrupted non-covalent interactions between native proteins and the ligand. Table 3 lists the ergosterol-interacting candidate proteins that were identified following LC-MS/MS according to functional categories, while proteins with low scores are presented in Table S1. The negative control (no ergosterol immobilized) protein list is given in Table S2.

#### 2.3.2. EAH Sepharose 4B Immobilized with Ergosterol-Hemisuccinate

**Figure 5:**
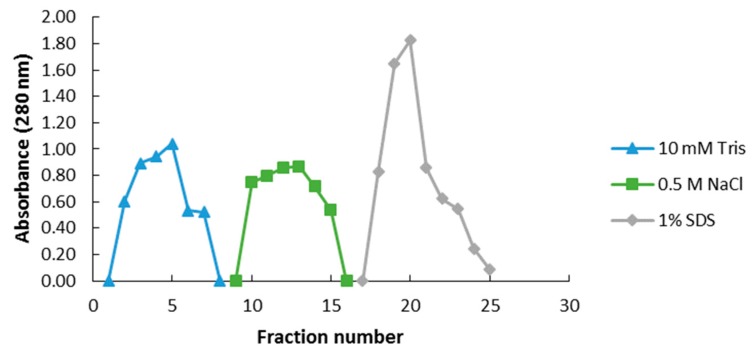
Representative elution profile of binding events between ergosterol-hemisuccinate immobilized on EAH Sepharose 4B resin and *A. thaliana* PM-associated proteins for the 6 h time point. The blue curve represents the flow-through fractions removed with 10 mM Tris-HCl, pH 7.5 buffer. The green curve represents the non-specifically bound fractions removed with 0.5 M NaCl in buffer, and the grey curve represents the proteins of interest eluted with 1% SDS in buffer.

**Figure 6:**
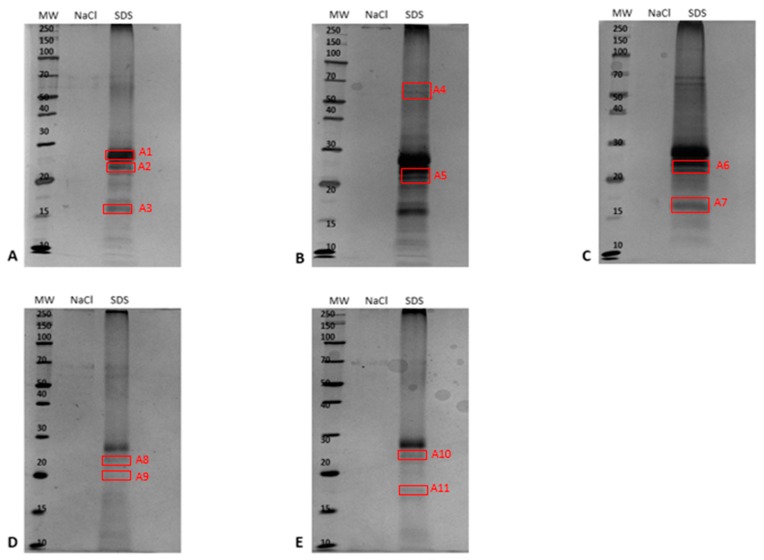
Comparative 12% 1D-SDS-PAGE analysis of ergosterol-interacting candidate proteins eluted with 0.5 M NaCl and 1% SDS during the affinity-capture procedure using EAH Sepharose 4B resin, where **A** = control, **B** = 0 h-, **C** = 6 h-, **D** = 12 h-, and **E** = 24 h-treated samples. For each fraction, 20 µg total protein was loaded and electrophoresed at constant 90 V at room temperature. The red blocks (A1–A11) were excised subsequent to silver staining and analyzed using LC-MS/MS for protein identification.

Ergosterol contains a diene group within its structure that is very reactive and requires protection by treatment with 4-phenyl-1,2,4-triazoline-3,5-dione (PTAD) prior to derivatization. Following protection, ergosterol was derivatized and validated using thin-layer chromatography (TLC) (shown in Figure S10). Figure 5 along with Figures S11–S15 show the binding events of the plasma membrane (PM)-associated fraction to the column immobilized with ergosterol-hemisuccinate. The NaCl and SDS fractions were analyzed using sodium dodecyl sulfate polyacrylamide gel electrophoresis (SDS-PAGE) and are illustrated in Figure 6. Selected bands were excised and analyzed using LC-MS/MS-based proteomics. The identified proteins are listed in Table 4, and proteins with low scores are in Table S3. The negative control (no ergosterol immobilized) protein list is presented on Table S4.

## 3. Discussion

### 3.1. Functional Classification of Identified Ergosterol-Responsive – and Interacting Candidate PM-Associated Proteins from A. thaliana Leaf Tissue

The PM is known to participate in a wide spectrum of important functions, including transport of ions across the membrane, communication with the extracellular environment, cell wall biosynthesis, and defense against invading microorganisms. These functions are achieved by transport and membrane trafficking proteins and receptor kinases [30,31,32]. As seen with most biochemical processes, proteins are not limited to one functional group, e.g., a transport protein may also be regulated during a defense response event. Such proteomic approaches (prior to enrichment and subsequent to affinity-based strategies) aimed to provide a comprehensive understanding of both ergosterol-responsive and interacting candidate proteins at the PM-localized interface, as well as those possibly associated with the PM subsequent to MAMP treatment.

#### 3.1.1. Membrane Trafficking and Transporters

In a plant cell, responses to a MAMP occurs within minutes [33]. As mentioned, ergosterol treatment causes ion fluxes across the PM and intracellular increase of Ca^2+^ levels. These changes are due to transport proteins and those involved in endocytosis/exocytosis. Aquaporins, identified in Table 1, Table 3, and Table 4 (i.e., both non-enriched and enriched PM-associated fractions), are water carrier proteins identified within all the time study samples and are also considered as PM markers. The PM intrinsic proteins (PIP) were differentially regulated in the samples, likely due to a defense response, and isolated during affinity chromatography. The *Arabidopsis* aquaporin AtPIP1 and AtPIP2 groups are well-known to be localized in PMs and are involved in defense responses within the plant [34].

ATP-dependent binding cassette (ABC) transporters have previously been shown to be involved in various processes such as transport of phytohormones, surface lipid deposition, and pathogen response during plant-microbe interactions [35]. In this study, an ABC transporter was identified in Table 3 (enriched PM-associated fraction) following capture affinity. The G family (AtABCG) group is the largest subfamily of ABC transporters in *A. thaliana,* and evidence was found by Ji et al. [36] that AtABCG16 is involved in basal resistance and abscisic acid (ABA) tolerance against the virulent bacterial pathogen *Pseudomonas syringae* pv. *tomato* (Pst) DC3000. Additionally, patellin-1 (Table 1, non-enriched PM-associated fraction) is a carrier protein involved in membrane trafficking by binding to hydrophobic molecules (such as the steroid-like ergosterol) and promoting their transfer between different cellular sites [37,38]. Vilakazi et al. [39] also identified this phosphoinositide-binding protein, patellin-1, in the study of capturing LPS-binding PM-associated proteins in *A. thaliana*.

Lastly, clathrin-dependent membrane trafficking is critical for determining cell polarity, and clathrin light chains are predominantly localized at the PM and early endosome compartments [40]. Both light chains (CLCs) and clathrin heavy chains (CHCs), including CHC1 and CLC3, were identified in the non-enriched PM-associated fraction (Table 1 and Table 2). In this regard, Mgcina et al. [41] also speculated that the binding-site of lipopolysaccharide (LPS) as a bacterial MAMP to *A. thaliana* protoplasts is internalized into the cell by endocytosis, thus leading to the reduced level of receptors on the surface. 

#### 3.1.2. Signaling

Pathogens that successfully overcome the initial physical defense barrier are mostly recognized by PRR proteins on the cell membrane. Recognition at the PM is immediately transmitted internally to activate other defense factors [42]. Some of the proteins involved during basal resistance fall within the signaling category and are associated with the PM during a defense response event. Here, a GPI-anchored protein was identified in the 12 h- and 24 h-treated PM-associated samples, as listed in Table 3 of the enriched fractions. These proteins are known to exist independently in a soluble form and are also associated with the PM [43]. GPI anchoring acts as a PM targeting signal, either in a localized or a polarized manner, by transferring signals from activated transmembrane receptors to various constituents inside the cell [44]. Due to these targeting mechanisms, GPI-anchored proteins are associated with lipid rafts/microdomains [43,45,46] and, since Peskan et al. [44] found evidence for such rafting in plants, these proteins have been used as a model or marker for raft sorting [47]. 

A LRR protein kinase-like protein and LRR-containing protein were identified in non-enriched as well as enriched PM-associated proteins (Table 1 and Table 3). LRR-containing RLKs are well known to confer resistance to bacterial and fungal pathogens [48]. A well-studied LRR-containing receptor is the FLS2 from *A. thaliana* that perceives the bacterial flagellin and triggers the binding of brassinosteroid intensive 1 (BRI1)-associated kinase (BAK1) to the receptor and acts as a signal enhancer [49]. Furthermore, FLS2 is said to migrate to highly organized membrane raft compartments of the PM where interaction with BAK1 takes place, forming a heterodimer [50]. Another protein kinase identified includes the G-type lectin S-receptor-like serine/threonine-protein kinase (GsSRK) listed in Table 1 (non-enriched fraction). Sanabria et al. [51] proposed a role for S-domain RLKs in M/PAMP perception, specifically for LPS. In the study, it was shown that LPS perception transiently up-regulates the expression of a G-type lectin receptor kinase in tobacco. This was also seen in the study of LPS perception in *A. thaliana* by Baloyi et al. [52], as the GsSRK protein was up-regulated during the time study. Plant lectins are proteins that are known to reversibly bind carbohydrates and are assumed to play a role in plant resistance and development. It was shown by Esch and Schaffrath [53] that the lectin domain of a jacalin-related lectin protein was responsible for relocating the protein towards the site of pathogen attack, and jacalin-related lectin 35 was identified in the 2D set of proteins analyzed subsequent to isolation (Table 2). The 14-3-3-like protein, GF14 epsilon, was also identified in the non-enriched fraction (Table 1). These proteins are known to be important components in biological pathways involved in signal transduction in response to biotic and abiotic stresses. In rice, 14-3-3 proteins regulate complex defense responses and interact with cellular components; 14-3-3 genes have also been found to be expressed in response to inoculation with rice fungal pathogens, thus suggesting functions in defense signaling [54].

CERK1 is a PM protein with three LysM motifs in the extracellular domain that was identified following affinity-capture (Table 3). LysM proteins have been shown to play a vital role in basal immunity by recognizing peptidoglycan and chitin via the *N*-acetylglucosamine (GlcNAc) moiety [52]. The *Arabidopsis* CERK1 (AtCERK1) is said to function as a ligand-binding protein and as a signaling molecule with kinase activity [55]. Lastly, the binding partner of accelerated cell death (ACD) 11 was identified for the first affinity-based approach (Table 3) and is known to mediate sphingolipid metabolism and regulate programmed cell death (PCD) upon pathogen infection in plants [56]. It has also been shown that *Arabidopsis* ACD mutant plants displayed excessive cell death upon infection with bacterial *P. syringae* [57].

#### 3.1.3. Defense responses

During the early stages of M/PTI, upon pathogen recognition, defense-related proteins are either activated, enhanced, or transcribed. Microbes can also deliver effectors into the cytosolic space of the plant cell during ETI, thus challenging the plant’s defense proteins [50]. The NDR1/HIN1-like protein 3 (NHL3), listed in the enriched fraction (Table 3), is predicted to be a membrane protein that has been shown to be triggered by avirulent *Pst* instead of the virulent strains. Hitherto, Varet et al. [58] reported that the expression of NHL3 is suppressed by virulent bacteria, and therefore the protein is hypothesized to participate in disease resistance. SNARE (soluble *N*-ethylmaleimide sensitive factor attachment protein receptors) complexes are also known to be necessary for immune responses and have been associated with targeted exocytosis of various antimicrobial compounds and proteins. Multiple SNARE complex constituents have been identified in previous studies, including the syntaxin of plants 122 (SYP122), and soluble *N*-ethylmaleimide-sensitive factor adaptor protein 23 (SNAP33) was identified in the 2DE samples (Table 2) in this study. These proteins were previously found to be highly enriched at the PM during an immune response [59].

Remorins, identified during the affinity-capture (Table 3), are proteins that play a role in cell-to-cell signaling and plant defense and have been shown to be associated with the PM in potato leaves. Furthermore, remorin 1.2 from tobacco (NtREM1.2) revealed primary accumulation in isolated DRMs and showed distinct localization in domains in the PM when expressed as a green fluorescent protein (GFP) fusion protein. These experiments showed that remorins are marker proteins for DRMs in plants that form higher order oligomers, impacting the binding affinity to these microdomains [46,60]. In *A. thaliana*, remorins are differentially phosphorylated, and this event is dependent on the presence of the NBR-LRR resistance protein RPM1. This is triggered upon perception of various M/PAMPs [61]. Within the *Arabidopsis* genome genes named, *AtONB1, AtBON2,* and *AtBON3* (bonzai, also known as copine) were shown to be regulators of plant immunity. The identified ergosterol-interacting candidate BONZAI-2 (enriched fraction in Table 3) plays a role in suppressing programmed cell death and defense in plants during pathogen attack [62]. This was supported by Zhou et al. [63], where *Arabidopsis* and rice plants were inoculated with *Pst* DC3000, and the pathogen’s interaction with the plant was limited to the PM. Hypersensitive-induced response (HIR) proteins are found on the PM and interact with LRR proteins during a defense response. The *A. thaliana* AtHIR1, AtHIR2, AtHIR3, and AtHIR4, identified in both non-enriched and enriched fractions (Table 1, Table 2 and Table 3), are associated with the intracellular side of the PM and involved in the development of programmed cell death during pathogen attack [39]. Baloyi et al. [52] and Vilakazi et al. [39] identified HIR protein 1, HIR protein 2, HIR protein 3, and HIR protein 4 in their studies pertaining to LPS as a M/PAMP.

Lastly, plant disease resistance (R) proteins are quantitative and rate-limiting regulators. Disease resistance protein RPP8, listed in the non-enriched fraction (Table 1), has been seen to be up-regulated in response to multiple avirulent pathogens and by wounding. It is also suggested that RPP8 is connected to multiple pathways [64].

## 4. Materials and Methods

### 4.1. Plant Growth and Elicitor Treatment

For the study, *A. thaliana* seedlings were grown in Culterra^TM^ Germination Mix (Culterra, Johannesburg, South Africa) soil in trays placed in a plant growth room at 20–24 °C under a 12-h light/12-h dark cycle until mature. Plants were routinely watered and fertilized with 1:300 (*v/v*) diluted Nitrosol^TM^ Natural (Nitrosol, Manukau City, New Zealand). Mature plants with fully developed rosettes (~2 months old) were treated with 250 nM ergosterol (Sigma, Steinheim, Germany) during the day cycle using gentle pressure infiltration into the abaxial side of the leaves. An elicitor stock solution was prepared in absolute ethanol and diluted in dH_2_O to a working solution containing less than 0.2% ethanol, and elicitation included a time study of 0, 6, 12, and 24 h, respectively, in accordance with related citations with untreated plants as the control. To eliminate any variation, all experiments included 3 biologicals and 3 repeats of each experiment, including sequencing. The raw data files containing the most significant proteins (Section 4.7) were merged to produce Supplementary Data Sheets 1–4 and compile Table 1, Table 2, Table 3 and Table 4.

### 4.2. Small-Scale Isolation of the Plasma Membrane(PM)-Associated Fraction

The isolation protocol was taken from Giannini et al. [26] and modified to optimize the yield of isolated fractions. Approximately 20 g of leaf tissue was homogenized in 60 mL homogenizing buffer containing 250 mM sucrose (Merck, Darmstadt, Germany), 3 mM ethylenediaminetetraacetic acid (EDTA) (MerckDarmstadt, Germany), 10% (*v/v*) glycerol, 0.5% (*w/v*) poly(vinylpolypyrrolidine) (PVPP) (Sigma, St. Louis, MO, USA), 2 mM phenylmethane sulfonyl fluoride (PMSF) (Boehringer Mannheim, Mannheim, Germany), 15 mM β-mercaptoethanol (Sigma, St. Louis, MO, USA), 4 mM 1,4-dithiothreitol (DTT) (Fisher Chemicals, Loughborough, UK), 250 mM potassium iodide (KI) (Saarchem, Johannesburg, South Africa), and 70 mM tris(hydroxymethyl)aminomethane (Tris) (Merck, Modderfontein, South Africa) using an Ultraturax homogenizer. Homogenates (HM) were filtered through 2 layers of miracloth (Millipore/Merck, Darmstadt, Germany) and centrifuged at 6000× *g* for 4 min at 4 °C using a Beckman Coulter^TM^ Avanti^TM^ J-20 I centrifuge. Cell debris was discarded, and the supernatants were collected and centrifuged at 13,000× *g* for 25 min at 4 °C. After centrifugation, the supernatants were discarded, and the pellets were resuspended in 800 µL microsomal resuspension buffer containing 250 mM sucrose, 10% (*v/v*) glycerol, 1 mM DTT, and 1 mM PMSF. Five hundred µL of the microsomal fraction was layered onto a sucrose gradient containing 700 µL of 25% (*w/v*) and 38% (*w/v*) sucrose each to create a discontinuous gradient in 1 mM Tris-HCl, 1 mM EDTA, and 0.1 mM DTT, pH 7.2. The gradients with the microsomal fractions were centrifuged at 13,000× *g* for 1 h, after which the PM-associated fraction formed an interface within the gradient and was aspirated using a pipette and transferred into a new tube. To validate the successful isolation of the said fractions, MAPK Western blot analysis (Figure S1) and plasma membrane H^+^-ATPase assays were routinely conducted. 

### 4.3. Identification of PM-Associated Ergosterol-Responsive Candidate Proteins

Prior to affinity chromatography, SDS-PAGE was performed of the homogenates, microsomal, and PM-associated fractions in order to identify proteins that could be categorized as ergosterol-responsive candidates. The 12% 1D-SDS-PAGE gels were visualized using the Fairbanks staining protocol [65], and differentially (densitometrically) regulated protein bands were excised for identification. The PM-associated fractions also underwent 2D-SDS-PAGE with immobilized pH gradient (IPG) strips of narrow range (pH 4–7) in order to identify elecrophoretically distinct spots that could be responsive candidates to ergosterol treatment. Samples were prepared for the first dimension of separation with a concentration of 100 µg total protein. A final volume of 120 µL sample was prepared containing 2 µL 50% DTT, 1.3 µL ampholyte (Bio-Rad, Hercules, CA, USA), x µL sample, y µL urea buffer with trace amounts of bromophenol blue. The samples were loaded onto the Immobiline™ Reswelling Dry-strip tray, and non-linear IPG strips [pH 3–10 or 4–7, 7 cm ReadyStrip™ IPG, Bio-Rad, Hercules, CA, USA)] were gently laid on top of the sample with the gel side down. The strips with samples were overlaid with mineral oil to prevent drying, and the tray was covered with foil. The strips were left to hydrate overnight at RT. Following hydration, the strips were placed on the Etthan IPGphorII electrophoresis unit (Amersham Bioscience, Buckinghamshire ,UK) with the gel side facing up. Electrode wicks were soaked with dH_2_O and placed on opposite ends of the strips. Conditions for isoelectric focusing (IEF) included step 1 at 250 V for 15 min, step 2 at 4000 V for 1 h, and step 3 was 4000 V for 12,000 V/h. Once IEF was completed, strips were rinsed with dH_2_O to remove excess mineral oil and then with 1X tank buffer for 5 min. The strips were incubated in DTT equilibration buffer (0.8 g DTT, 6 M urea, 30% glycerol, 2% SDS, 50 mM Tris-HCl, pH 8.8) for 20 min with constant shaking. Strips were rinsed again with 1X tank buffer and then incubated in iodoacetamide (IAA) (Sigma, St. Louis, MO, USA) equilibration buffer (0.2 g IAA, 6 M urea, 30% glycerol, 2% SDS, 50 mM Tris-HCl, pH 8.8) for 20 min with constant shaking, followed by 1X tank buffer prior to loading the strip on top of a 12% resolving gel, as previously prepared. Protein spots that showed differential regulation were excised and analyzed by LC-MS.

### 4.4. Affinity Chromatography

#### 4.4.1. Magnetic Epoxide Microspheres

The MagReSyn^TM^ magnetic epoxide microspheres (ReSyn Biosciences, AEC-Amersham, Midrand, South Africa) were supplied as a 20 mg/mL suspension in 20% (*v/v*) ethanol. The microspheres contain high functional group intensity throughout the fiber surface network, which allows a ligand (in this case, ergosterol) to react and be immobilized to the lattice by covalent bonding [66]. Microspheres were resuspended in the shipping solution, and 50 µL (±1 mg) was collected with a pipette and transferred to a new tube. The tube was placed on a magnetic separator, and once microspheres were clear, the shipping solution was discarded. Microspheres were equilibrated with three washes of 200 µL milliQ H_2_O. The activation solution supplied by the manufacturer consisting of 5.2 M 1,4-butanediol diglycidyl ether was diluted 4× to a working solution. Microspheres were resuspended in 500 µL activation solution and continuously agitated for 48 h at RT before removal thereof and washing of the microspheres two times with 200 µL 90% (*v/v*) tetrahydrofuran (THF) (Sigma, Steinheim, Germany), which was also used as the coupling buffer. Thirty mg/mL of ergosterol was prepared with 90% (*v/v*) coupling buffer. Five hundred µL of the ergosterol was added to the activated microspheres and continuously agitated for 48 h at 4 °C. On the magnetic separator, the unbound ergosterol was removed, and microspheres were washed three times with 200 µL coupling buffer. Epoxide residues that did not bind to the ergosterol were quenched with 500 µL ethanolamine, pH 8.5 blocking solution for 24 h at RT. The blocking agent was discarded, and 1 mg/mL (~1.25 mL) of PM-associated protein sample was added. The microspheres with the samples were incubated for 24 h at RT with constant agitation followed by removal of the liquid fraction and washing of the microspheres five times with 1 mL 10 mM Tris-HCl, pH 7.5 to remove unbound proteins. The microspheres were then washed with 1 mL 0.5 M NaCl in 10 mM Tris-HCl, pH 7.5 to remove non-covalently bound proteins. Ergosterol-interacting candidate proteins were subsequently eluted with five washes 1 mL 1% (*w/v*) SDS in 10 mM Tris-HCl, pH 7.5. The absorbance of the collected fractions was measured spectrophotometrically at 280 nm for monitoring absorption and desorption reactions. 

#### 4.4.2. EAH Seharose 4B

This approach required the derivatization of ergosterol to the hemisuccinate for affinity chromatographic applications, as reported by Tejada-Simon and Pestka [67]. Thirty mg/mL ergosterol was treated with 4-phenyl-1,2,3-triazoline-3,5-dione (PTAD) (Sigma, Steinheim, Germany), resulting in cyclic adducts. For the formation of the adducts to ergosterol-hemisuccinate, 2.5 mM succinic anhydride (Sigma, Steinheim, Germany) was dissolved in 800 µL pyridine (Sigma, St. Louis, MO, USA) in a reaction vessel. The ergosterol adducts were added to the mixture and refluxed for 60 min. The reaction was allowed to cool down for ±5 min before the reaction vessel was submerged in boiling water and a nitrogen steam was applied to remove pyridine. Excess succinic anhydride was removed by portioning the mixture in equal volumes of water and chloroform. The chloroform phase containing ergosterol-hemisuccinate (Erg-HS) was dried under nitrogen. To authenticate the successful derivatization of ergosterol, 30 mg/mL ergosterol and 30 mg/mL Erg-HS were separately dissolved in toluene:acetone (70:30, *v/v*). High-performance thin layer chromatography (HP-TLC) was conducted with a mobile phase of toluene:acetone (70:30, *v/v*), and plates were visualized under UV at 254 nm.

One mL of 1,6-diaminohexane (EAH) Sepharose 4B beads were swollen in 10 mL 0.5 M NaCl and washed two times with 5 mL water, pH 4.5. Erg-HS (0.05 g) was dissolved in 2 mL 50% (*v/v*) 1,4-dioxane (Sigma, Steinheim, Germany) and added to the beads. One-ethyl-3(3-dimethylaminopropyl)-carbodiimide (Sigma, Steinheim, Germany) was added to the beads to a final concentration of 0.13 M and manually inverted for 60 min at RT. The carbodiimide-promoted condensation reaction was then continuously inverted for 24 h at 4 °C to allow the coupling of Erg-HS to the beads. This was followed by washing with 10 mL 100% 1,4-dioxane, 10 mL 80% (*v/v*) ethanol, 20 mL water, and 10 mM Tris-HCl pH 7.5, respectively. Beads were transferred into a column, 1 mg/mL of the PM-associated extract was added on the resin bed, and the column was blocked for 1 h at RT to allow binding of proteins to Erg-HS. The column was then washed six times with 50 mM Tris-HCl pH 7.5 to remove all unbound proteins followed by six washes with 0.5 M NaCl in 50 mM Tris-HCl 7.5 to remove non-specifically bound proteins. Lastly, ergosterol-interacting candidate proteins were eluted with 1% (*w/v*) SDS in 50 mM Tris-HCl pH 8.0. The absorbance of the collected fractions was spectrophotometrically measured at 280 nm to determine the elution profile.

### 4.5. Protein Precipitation and SDS-PAGE

Proteins from the desorbed affinity fractions were precipitated with absolute acetone at a 1:2 (*v/v*) ratio at −20 °C overnight to remove any substances that may have interfered with SDS-PAGE and mass-spectrometry and to achieve maximum protein yield. Fractions with acetone were briefly vortexed prior to centrifugation at 13,000× *g* for 10 min at 4 °C, after which the supernatant was carefully discarded. Pelleted proteins were washed twice with ice cold 80% (*v/v*) acetone, centrifuged at 13,000× *g* for 10 min between each wash step, and solubilized in SDS sample buffer. SDS-PAGE was performed by resolving ergosterol-interacting candidate proteins on 12% gels using the Hoefer Scientific miniVE vertical electrophoresis system at constant voltage of 90 V for 3 h at RT (Section 4.2). Gels were stained using the silver staining protocol adapted from Switzer III et al. [68] and Blum et al. [69].

### 4.6. In-Gel Trypsin Digestion

Coomassie-stained gel slices were destained twice in a solution containing 100 mM ammonium bicarbonate (NH_4_HCO_3_) (Sigma, Steinheim, Germany) and 50% (*v/v*) acetonitrile (ACN) for 45 min at RT with constant agitation, while silver-stained gel pieces were covered with a solution of 30 mM potassium ferricyanide and 100 mM sodium thiosulfate and agitated until clear at RT. Solutions were discarded, and gel pieces were washed with milliQ H_2_O followed by an addition of 2 mM Tris(2-carboxyethyl) phosphine (TCEP) (Sigma, Steinheim, Germany) that was made up in 25 mM NH_4_HCO_3_ and incubated for 15 min at RT with constant agitation to reduce proteins. Excess TCEP was removed, and gel pieces were washed three times with 500 µL NH_4_HCO_3_ for 15 min. Concentrated ACN was added, and samples underwent desiccation under vacuum centrifugation. Thereafter, gel pieces were covered with sequencing grade trypsin (20 ng/µL) (Promega, Madison, WI, USA) dissolved in 50 mM NH_4_HCO_3_ and incubated on ice for 1 h. Excess trypsin solution was removed, and sufficient 50 mM NH_4_HCO_3_ was added, followed by incubation for 18 h at 37 °C. Peptides were extracted from gel pieces using 0.1% (*v/v*) trifluoroacetic acid (TFA) (Sigma, St. Louis, MO, USA) and incubation for 1 h at 37 °C. Peptides were then collected by centrifugation at 40× *g* for 5 min and dried under vacuum, followed by resuspension in 12 µL loading buffer [2% ACN, 0.1% formic acid (FA) (Sigma, St. Louis, MO, USA)] prior to analysis.

### 4.7. LC-MS/MS Analysis

Analysis was conducted at the Centre for Proteomics and Genomic Research (CPGR) (Cape Town, South Africa). Nano-RP LC was performed on a Dionex Ultimate 3000 nano-HPLC system coupled with a Q-Extractive Quadrupole-Orbitrap mass spectrometer (Thermo Fisher Scientific, Waltham, MA, USA) for LC-MS/MS analysis. The mobile phase solvent system employed was solvent A: water/0.1% FA and solvent B: 100% ACN/0.1% FA. Solubilized peptides were loaded onto a C18 trap column (300 µm × 5 mm × 5 µm). Separation was performed on a C18 column (75 µm × 20 cm × 1.7 µm), and a linear gradient was generated at 300 nL/min with a change of 2–60% solvent B over 52 min. The mass spectrometer was operated in positive ion mode with a capillary temperature of 320 °C. The applied electrospray voltage was 1.95 kV. Lastly, mass spectrometry was performed using data-dependent acquisition MS/MS scans with a mass range of 350–2000 *m/z*.

### 4.8. Data analysis

Data analysis was performed using the Byonic software (Protein Metrics, Cupertino, CA, USA), product version PMI-Byonic-Com: v2.6.46. The Arabidopsis UniProt Knowledgebase (UniprotKB) database [70] was used to match peptide fragments resulting from the MS/MS. Peptides were fragmented using the collision-induced dissociation (CID) low energy, and the parameters were as follows: trypsin was a C-terminal cutter end, and carbamidomethyl (C) and deamination (NQ) were set for fixed and variable modification, respectively. The precursor tolerance was 7 ppm, and the fragment tolerance was 20 ppm. Maximum number of missed cleavage was 2, and the protein false discovery rate (FDR) cut-off was 1% with the best score range of 0–1000 *m/z*. A target protein with a best score of >300 was considered significant.

## 5. Conclusions

The purpose of the study was to identify *Arabidopsis* PM-associated candidate proteins in the response to ergosterol treatment as well as those possibly interacting with the MAMP as ligands. A small-scale isolation protocol was used to fractionate the *Arabidopsis* leaf tissue and resulted in the successful isolation of said fraction for different time points. This was confirmed by identification of PM and DRM markers subsequent to LC-MS/MS-based proteomics in excised 1D- and 2D-SDS-PAGE protein bands and spots from the non-enriched fraction (i.e., responsive candidate proteins). Thereafter, enrichment of PM-associated proteins (i.e., ergosterol-interacting candidate proteins) resulted in some that had been identified in previous studies using different elicitors, such as the bacterial flg22 and LPS, as well as fungal chitin. The perception mechanism of ergosterol in *Arabidopsis* is still unclear, but the identified candidate proteins show that there could possibly be a receptor complex (including non-PM yet associated proteins) involved in signaling the recognition of this MAMP to the intracellular components of the plant cell, and that is similar to other reported elicitors. Additionally, the second affinity-enrichment, which was meant to reduce non-specific binding, yielded very few ergosterol-interacting PM-associated candidate proteins, and this may suggest that derivatization of ergosterol resulted in critical alterations to molecular features that could have affected the association/interaction of the MAMP molecule with proteins.

Lastly, this study is a first of its kind because affinity chromatography has not yet been employed for capturing ergosterol-interacting PM-associated candidate proteins. As previously mentioned, the PM is the recognition site of many microorganisms and associated MAMPs, therefore understanding or identifying the interface proteomic changes involved during ergosterol-induced MTI may assist in further elaborating the protein network and pathways regulated during activation of immune responses that form part of plant defense.

## Figures and Tables

**Table 1:** LC-MS/MS identification of *A. thaliana* PM-associated responsive proteins from selected 1D SDS-PAGE bands of control, 0-, 6-, 12-, and 24 h fractions subsequent to ergosterol treatment and organized according to functional categories (Supplementary Data Sheet 1).

Sample No.	Protein Name	Accession No.	Biological GO Term	Molecular GO Term	Calculated Mass ^a^ (M + H)	Mass Error ^b^ (ppm)	Byonic ™ Score ^c^	|Log Prob|^d^
**Perception and signaling (17)**								
A5	Calcium-dependent lipid-binding (CaLB domain) family protein At3g61050	Q9LEX1	Response Signaling	DNA-binding	1214.699	−0.6	422.1	8.18
A7	Non-lysosomal glucosylceramidase At4g10060	F4JLJ2	Lipid Metabolism	Glycosidase	1294.627	−1.9	395.8	7.88
A10	G-type lectin S-receptor-like serine/threonine-protein kinase CES101 At3g16030	Q9LW83	Perception Response	Transferase	1113.626	−0.6	350.0	3.23
A5	Nicalin At3g44330	Q9M292	Signaling	---	1142.642	0.4	335.6	5.34
A7, A12	Cysteine-rich receptor-like protein kinase 41 At4g00970	O23081	Signaling	Transferase	973.531	0.3	328.0	1.53
A3	Axi 1 protein-like protein At2g44500	O64884	Biosynthesis Metabolism	Transferase	928.535	−2.9	289.7	2.72
A7	Cysteine-rich receptor-like protein kinase 10 At4g23180	Q8GYA4	Signaling	Transferase	1223.667	0.0	285.9	6.63
A7	PQQ_DH domain-containing protein At5g11560	F4JXW9	Biosynthesis	---	992.541	1.2	251.0	5.58
A8	Probable serine/threonine-protein kinase At4g35230	Q944A7	Defense	Transferase	1269.741	−2.3	236.2	6.31
A4	14-3-3-like protein GF14 epsilon At1g22300	P48347	Signaling	Protein binding	1229.580	−1.5	230.0	5.62
A9	Phosphoinositide phospholipase C 2 At3g08510	Q39033	Defense	Hydrolase	996.645	−0.5	228.4	4.97
A7	AMP deaminase At2g38280	O80452	Response	Hydrolase	1123.563	0.9	224.0	4.69
A10	Probable inactive leucine-rich repeat receptor-like protein kinase At3g03770	Q8LFN2	Signaling	Kinase	1041.515	0.4	217.2	1.30
A13	Mitogen-activated protein kinase 8 At1g18150	Q9LM33	Signaling	Kinase	1028.537	0.4	200.2	8.87
A7	Putative leucine-rich repeat receptor-like serine/threonine-protein kinase At2g24130	Q9ZUI0	Signaling	Transferase	1149.626	2.2	174.2	1.02
A7	Leucine-rich repeat receptor-like protein kinase At2g01210	Q9ZU46	Signaling	Transferase	870.541	0.1	164.6	0.9
A7	Receptor-like kinase TMK4 At3g23750	Q9LK43	Signaling	Kinase	1020.572	0.6	121.5	1.15
**Membrane trafficking and transport (16)**								
A5	V-type proton ATPase subunit B2 At4g38510	Q9SZN1	Transport	Hydrolase	1563.801	−1.4	574.5	9.38
A7	Patellin-1 At1g72150	Q56WK6	Growth	Lipid binding	1231.689	−0.7	515.8	7.93
A3	Ras-related protein RABE1c At3g46060	P28186	Signaling Transport	GTPase	1071.641	−0.9	412.5	8.36
A7	ATPase 1, plasma membrane-type At2g18960	P20649	Transport	Translocase	1040.574	0.5	401.7	7.98
A6	Ras-related protein RABA1g At3g15060	Q9LK99	Signaling Transport	GTPase	1043.610	−0.1	384.7	8.14
A7	Clathrin heavy chain 1 At3g11130	Q0WNJ6	Transport	Clathrin binding	992.578	0.5	289.6	5.65
A3, A7	Probable aquaporin PIP1-5 At4g23400	Q8LAA6	Transport	Water transport	1049.599	−0.5	288.9	6.62
A5	Aquaporin PIP1-2 At2g45960	Q06611	Transport	Water transport	1033.604	−0.7	282.3	6.57
A7	CSC1-like protein ERD4 At1g30360	Q9C8G5	Transport	Ion channel	1251.612	0.5	271.5	7.51
A4	Probable ADP, ATP carrier protein At5g56450	Q9FM86	Transport	ATP:ADP transport	1021.531	−0.3	254.1	5.24
A3	Ras-related protein RABA1e At4g18430	O49513	Signaling Transport	GTPase	1274.612	−1.4	240.9	7.40
A8	Aquaporin TIP1-2 At3g26520	Q41963	Transport	Water transport	1980.030	0.0	239.9	6.69
A4, A5, A8	Aquaporin PIP2-1 At3g53420	P43286	Transport	Water transport	1069.568	0.2	215.3	5.98
A5	Probable aquaporin PIP2-6 At2g39010	Q9ZV07	Transport	Water transport	1311.669	−0.8	214.5	1.65
A7	Exocyst complex component SEC3A At1g47550	Q9SX85	Transport	GTP-Rho binding	1015.578	−1.7	183.9	1.26
A1	Aluminum-activated malate transporter 6 At2g17470	Q9SHM1	Transport	Malate transporter	1606.832	2.8	40	1.29
**Defense (6)**								
A5	Trans-cinnamate 4-monooxygenase At2g30490	P92994	Biosynthesis Defense	Monooygenase activity	1271.721	−0.3	377.3	8.02
A9	Protein BONZAI 2 At5g07300	Q5S1W2	Response	Phospholipid binding	1199.663	0.2	340.2	7.66
A3	Temperature-induced lipocalin-1 At5g58070	Q9FGT8	Response	Storage protein	1110.531	−0.5	329.0	7.88
A7	Disease resistance protein RPP8 At5g43470	Q8W4J9	Defense	ATP:ADP binding	1140.557	−2.1	267.7	6.39
A4	Hypersensitive-induced response protein 3 At3g01290	Q9SRH6	Response	---	949.547	−1.6	237.0	5.91
A4	Uncharacterized protein (LOW PSII ACCUMULATION-like protein) At4g28740	F4JM22	Chloroplast	---	995.600	-0.1	131.8	1.24
**Structure (1)**								
A2	Putative clathrin assembly protein At1g14910	P94017	Transport	Clathrin binding	1314.742	−1.3	122.2	1.22
**Unknown (8)**								
A11	Triacylglycerol lipase-like 1 At1g45200	Q8L7S1	Metabolism	Hydrolase	1222.622	−0.2	336.6	6.54
A11	TNF receptor associated factor (TRAF)-like family protein At1g58270/F19C14_8	Q9SLV3	Signal transduction	----	1434.722	−0.8	286.2	6.92
A7	Uncharacterized protein At4g16180	F4JLQ2	---	---	1293.669	−2.2	235.3	5.38
A1	Putative uncharacterized protein At3g19340	Q8RWC3	---	Aminopeptidase	1219.632	−0.9	229.5	5.94
A12	Putative uncharacterized protein F14P22.240 At3g58650	Q9M2F2	Growth	---	472.288	−1.1	160.4	1.04
A12	Putative uncharacterized protein F3A4.21 At3g50130	Q9SN05	---	---	472.288	−1.1	160.4	0.92
A2	Uncharacterized protein At4g38260	F4JTM0	---	---	1245.520	−5.2	142.3	0.98
A2	EMB|CAB72473.1 At5g22560	Q9FK83	---	---	1467.731	0.1	133.6	1.21

a = the computed M + H precursor mass for the peptide spectrum matches (PSMs); b = a calculated mass error (parts per million) after correcting the observed M + H (single charged) precursor mass and the computed M + H precursor mass; c = Byonic score, and primary indicator of PSM correctness. A score of 300 is considered to be a significant hit [29]; d = the log p-value of the PSM, of which the value should be ≥ 1 for a hit to be significant. Proteins highlighted in red are known plasma membrane (PM) markers.

**Table 2:** LC-MS/MS identification of *A. thaliana* PM-associated responsive proteins from selected 2D SDS-PAGE spots of control, 0- , 6- , 12-, and 24 h fractions subsequent to ergosterol treatment and arranged according to functional categories (Supplementary Data Sheet 2).

Sample No.	Protein Name	Accession No.	Biological GO Term	Molecular GO Term	Calculated Mass ^a^ (M + H)	Mass Error ^b^ (ppm)	Byonic ™ Score ^c^	|Log Prob| ^d^
**Perception and signaling (10)**								
B7	Probable serine/threonine-protein kinase At4g35230	Q944A7	Signaling	Transferase	1269.741	0.1	480.6	7.29
B4	At2g34560 protein (P-loop containing nucleoside triphosphate hydrolase) At2g34560	B9DGC0	Transport	ATPactivity	1156.672	−0.8	401.3	8.95
B3	Aspartyl aminopeptidase At5g60160/f15|12_20	Q9LST0	Biosynthesis	Metalloaminopeptidase	1148.679	0.3	382.0	8.65
B8	Probable protein phosphatase 2C 20 At2g20630	Q9SIU8	Signaling	Hydrolase	1288.711	−0.1	363.1	7.51
B6	Abscisic acid receptor PYL1 At5g46790	Q8VZS8	Signaling	Receptor	1442.760	−0.5	357.2	9.01
B7	Phosphotidylinositol 4-kinase alpha 1 At1g49340	Q9SXA1	Signaling	Kinase	1964.041	0.1	345.5	6.85
B2	Protein SGT1 homolog B At4g11260	Q9SUT5	Signaling	---	1435.709	−1.4	351.9	8.86
B1	Fasciclin-like arabinogalactan protein 7 At2g04780	Q9SJ81	Biosynthesis	---	981.500	0.9	322.0	6.49
B2	1-Phosphotidylinositol-3-phosphate 5-kinase FAB1A At4g33240	Q0WUR5	Signaling	Kinase	1470.816	−1.8	303.8	7.75
B8	Plasma membrane-associated cation-binding protein 1 At4g20260	Q96262	Response	Ion binding	1146.641	0.0	281.2	7.00
**Membrane trafficking and transport (16)**								
B2	V-type proton ATPase subunit B3 At1g20260	Q8W4E2	Transport	Hydrolase	1563.801	−1.9	442.2	9.76
B8	Alpha-soluble NSF attachment protein 2 At3g56190	Q9SPE6	Transport	---	1259.684	−0.8	426.3	8.35
B6	Ras-related protein RABA1d At4g18800	Q9SN35	Signaling	GTPase	1043.610	0.0	414.7	7.96
B4, B7	Patellin-2 At1g22530	Q56ZI2	Transport	Lipid-binding	1520.784	−0.7	391.9	9.24
B4, B7	Patellin-1 At1g72150	Q56WK6	Transport	Lipid-binding	1231.689	−2.0	372.2	5.53
B2	Clathrin light chain 3 At3g51890	F4J5M9	Transport	Clathrin binding	855.530	0.0	363.4	4.96
B6	Ras-related protein RABA5b At3g07410	Q9SRS5	Signaling	GTPase	1071.641	−0.9	357.3	8.16
B3	SNAP25 homologous protein SNAP33 At5g61210	Q9S7P9	Transport	SNAP receptor	1302.715	−1.3	352.6	6.82
B1, B4, B8	V-type ATPase catalytic subunit A At1g78900	O23654	Transport	Hydrolase	1019.552	−1.6	338.6	5.50
B7	Sugar transport protein 7 At4g02050	O04249	Transport	Transmembrane transporter	1006.469	0.9	338.1	6.27
B3	Auxin transport protein BIG At3g02260	Q9SRU2	Signaling	Zinc binding	589.356	−1.4	331.2	5.39
B3	Protein NETWORKED 1C At4g02710	Q9ZQX8	---	Actin binding	478.251	0.1	311.4	6.18
B3	ABC transporter C family member 8 At3g21250	Q8LGU1	Transport	Translocase	530.330	−0.3	303.2	5.82
B8	Syntaxin-71 At3g09740	Q9SF29	Transport	SNAP receptor	1081.636	0.7	299.7	7.30
B4, B7	Flotillin-like protein 1 At5g25250	Q501E6	Transport	---	1526.909	−1.8	273.6	6.93
**Defense (9)**								
B2, B4, B7	Jacalin-related lectin 35 At3g16470	O04309	Perception Response	Carbohydrate binding	1469.763	−2.5	518.7	9.21
B6	Aluminium induced protein with YGL and LRDR motifs At5g19140	Q94BR2	---	---	1439.738	−1.6	420.4	8.77
B6, B8	At3g11930 protein (Adenine nucleotide alpha hydrolases-like) At3g11930	B9DG73	---	Hydrolase	1189.631	−2.2	380.6	8.02
B4	Callose synthase 9 At3g07160	Q9SFU6	Biosynthesis Defense	Transferase	557.402	−0.9	334.8	2.44
B8	Hypersensitive-induced response protein 4 At5g51570	Q9FHM7	Defense Signaling	---	1466.764	1.7	345.6	8.36
B8	Binding partner of ACD (accelerated cell death)11 1 At5g16840	Q9LFD5	Signaling	RNA-binding	1132.621	−1.0	332.1	7.69
B5, B8	Hypersensitive-induced response protein 2 At1g69840	Q9CAR7	Defense Signaling	Kinase binding	871.500	−1.3	327.2	4.57
B6	Dessication responsive protein At2g21620	Q94II5	---	Hydrolase	980.614	−0.8	293.5	6.84
B8	Hypersensitive-induced response protein 1 At5g62740	Q9FM19	Defense Signaling	Kinase-binding	949.547	−0.5	281.6	7.29

a = the computed M + H precursor mass for the peptide spectrum matches (PSMs); b = a calculated mass error (parts per million) after correcting the observed M + H (single charged) precursor mass and the computed M + H precursor mass; c = Byonic score, and primary indicator of PSM correctness. A score of 300 is considered to be a significant hit [29]; d = the log p-value of the PSM, of which the value should be ≥ 1 for a hit to be significant. Proteins highlighted in red are known PM markers.

**Table 3:** LC-MS/MS identification of *A. thaliana* PM-associated candidate proteins interacting with ergosterol immobilized on epoxide magnetic microspheres for control, 0-, 6-, 12-, and 24 h subsequent to treatment and listed according to functional categories (Supplementary Data Sheet 3).

Sample No.	Protein Name	Accession No.	Biological GO Term	Molecular GO Term	Calculated Mass ^a^ (M + H)	Mass Error ^b^ (ppm)	Byonic™ Score ^c^	|Log Prob|^d^
**Signaling**								
A12	Uncharacterized glycosylphophatidylinositol (GPI)-anchored protein At5g19250	P59833	---	---	1910.898	−3.1	464.4	8.69
A13	Binding partner of ACD (accelerated cell death)11 1 At5g16840	Q9LFD5	Signaling Response	RNA-binding	1132.621	−1.0	428.4	8.21
A4	Probable inactive receptor kinase At3g02880	Q9M8T0	Response	Receptor	1426.706	−3.0	392.2	6.99
A13	Uncharacterized GPI-anchored protein At5g19250	P59833	---	---	1910.898	−3.5	388.2	7.32
A11	Leucine-rich repeat-containing protein At5g07910	Q8RWI2	Response	---	1269.727	−0.5	336.8	7.18
A4	Probable inactive receptor kinase At5g16590	Q9FMD7	Response	Receptor	2127.170	−1.5	324.5	8.27
A11	Leucine-rich repeat protein kinase-like protein At1g10850	Q940B9	Signaling Response	Kinase	984.584	1.0	322.0	6.56
A11, A14	Chitin elicitor receptor kinase 1 At3g21630	A8R7E6	Perception Signaling	Kinase	1132.596	−0.6	313.8	6.87
**Membrane trafficking and transport**								
A1, A5	Aquaporin PIP2-7 At4g35100	P93004	Transport	Water channel	1312.653	−1.4	541.6	7.44
A1	Aquaporin PIP1-2 At2g45960	Q06611	Transport	Water channel	1017.548	−0.3	509.0	6.59
A1	Aquaporin PIP2-1 At3g53420	P43286	Transport	Water channel	2000.996	−1.2	473.2	10.44
A1	Probable aquaporin PIP1-5 At4g23400	Q8LAA6	Transport	Water channel	1230.632	−1.1	464.3	9.05
A8	Plasma membrane-associated cation-binding protein 1 At4g20260	Q96262	Response	Ion-binding	1425.711	−2.6	418.8	8.56
A4	ATPase 2, plasma membrane-type At4g30190	P19456	Transport	Translocase	1040.574	−0.2	412.0	7.60
A6	At2g34250 protein At2g34250	O80774	Transport	Protein transport	1164.601	−2.0	408.2	6.50
A1	Ras-related protein RABE1c At3g46060	P28186	Signaling	GTPase	1071.641	−0.7	394.8	8.40
A9, A12	CASP-like protein 1D1 At4g15610	Q9FE29	---	---	1127.657	−0.3	389.4	9.41
A11	Plasma membrane ATPase At4g30190	F4JPJ7	Transport	Translocase	1040.574	−0.2	381.3	8.04
A11	ATPase 5, plasma membrane-type At2g24520	Q9SJB3	Transport	Translocase	1040.574	0.0	362.8	7.00
A11	Fasciclin-like arabinogalactan protein 8 At2g45470	O22126	---	---	967.484	−0.8	348.8	7.71
A11	Patellin-1 At1g72150	Q56WK6	Transport	Lipid-binding	1078.589	−1.2	326.8	7.67
A1	F-box/LRR-repeat protein At3g60040	Q8GWI2	Response	---	784.529	−0.3	309.1	1.05
A9	ABC transporter G family member 41 At4g15215	Q7PC83	Transport	ATP-binding	543.386	−0.9	301.8	4.38
A6	CSC1-like protein ERD4 At1g30360	Q9C8G5	Transport	Ion channel	1583.850	2.7	301.4	7.40
**Defense response**								
A5	Hypersensitive-induced response protein 3 At3g01290	Q9SRH6	Defense Signaling Response	---	1519.775	−1.6	489.0	8.32
A6	Syntaxin-121 At3g11820	Q9ZSD4	Defense	SNAP receptor	1329.701	−1.4	439.9	7.40
A10	NDR1/HIN1-like protein 3 At5g06320	Q9FNH6	Defense	---	1496.843	−1.8	394.3	8.73
A10	Protein BONZAI 2 At5g07300	Q5S1W2	Defense	Phospholipid-binding	1060.615	0.0	372.4	7.25
A11, A14	Remorin At2g45820	O80837	---	---	617.409	−1.8	358.2	4.82
A11, A14	Blue copper protein At5g20230	Q07488	Transport	Electron transfer	1425.664	−0.4	337.8	9.34
**Unknown**								
A11	At1g55160/T7N22.11 At1g55160	Q9C542	---	---	1174.631	−0.2	384.7	8.38
A11	At3g08600/F17014_7 At3g08600	Q9C9Z6	---	---	903.453	−0.2	352.4	7.93
A14	Expressed protein At2g18690	Q9ZV49	---	---	1115.606	−1.0	319.2	6.03

a = the computed M + H precursor mass for the peptide spectrum matches (PSMs); b = a calculated mass error (parts per million) after correcting the observed M + H (single charged) precursor mass and the computed M + H precursor mass; c = Byonic score, primary indicator of PSM correctness. Score of 300 is considered to be a significant hit [29]; d = the log p-value of the PSM, which the value should be ≥ 1 for hit to be significant.

**Table 4:** LC-MS/MS identification of *A. thaliana* PM-associated candidate proteins interacting with ergosterol-hemisuccinate immobilized on EAH Sepharose 4B resin for the time study (Supplementary Data Sheet 4).

Sample No.	Protein Name	Accession No.	Biological GO Term	Molecular GO Term	Calculated Mass ^a^ (M + H)	Mass Error ^b^ (ppm)	Byonic™ Score ^c^	|Log Prob|^d^
A7	Ras-related protein RABG1 At5g39620	Q948K6	Signaling	GTP-binding	1071.641	0.0	515.00	6.92
A4	Aquaporin PIP1-2 At2g45960	Q06611	Transport	Water channel	1033.604	−1.2	336.3	6.55
A10	1-Phosphotidylinositol-3-phosphate-5-kinase FAB1B At3g14270	Q9LUM0	---	Kinase	956.480	−0.5	328.4	6.19
A6	Ras-related protein RABE1c At3g46060	P28186	Signaling	GTP-binding	1164.590	0.6	319.2	6.34
A1	Aquaporin PIP2-1 At3g53420	P43286	Transport	Water channel	1069.568	0.4	284.5	5.82

a = the computed M + H precursor mass for the peptide spectrum matches (PSMs); b = a calculated mass error (parts per million) after correcting the observed M + H (single charged) precursor mass and the computed M + H precursor mass; c = Byonic score, primary indicator of PSM correctness. Score of 300 is considered to be a significant hit [29]; d = the log p-value of the PSM, which the value should be ≥ 1 for hit to be significant.

## Supplementary Materials

Supplementary materials can be found at https://www.mdpi.com/1422-0067/20/6/1302/s1.
