# Supplementary material for: Identification of Candidate Ergosterol-Responsive Proteins Associated with the Plasma Membrane of Arabidopsis thaliana
Source: Int J Mol Sci. 2019 Mar 14;20(6):1302. doi: 10.3390/ijms20061302 (PMC6471938; doi:10.3390/ijms20061302)
Supplement: Supplementary file 1 [file ijms-20-01302-s001.pdf]

International Journal of
Molecular Sciences

MDPI

Article

Analysis of candidate ergosterol-responsive and interacting proteins associated with the plasma membrane of *Arabidopsis thaliana*

Thembisile G. Khoza, Ian A. Dubery and Lizelle A. Piater*

Department of Biochemistry, University of Johannesburg, Auckland Park, 2006, South Africa; tkhoza03@gmail.com (T.K); idubery@uj.ac.za (I.D.)
* Correspondence: lpiater@uj.ac.za; Tel.: +27-11-559-2403

Received: date; Accepted: date; Published: date

Supplementary Figures

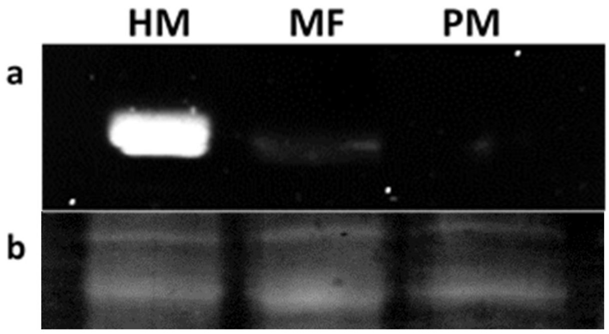

Figure S1: Representative Western blot analysis for (**a**) *Arabidopsis thaliana* MAPKs (probed with anti-active MAPK pAb, rabbit (pTEpY) (Promega, USA)) in the isolated homogenate (HM), microsomal fraction (MF) and plasma membrane (PM-associated) subsequent to 6 h ergosterol treatment and (**b**) an Amido Black PVDF-stained loading control showing that lack of MAPK activity is not due to absence of proteins.

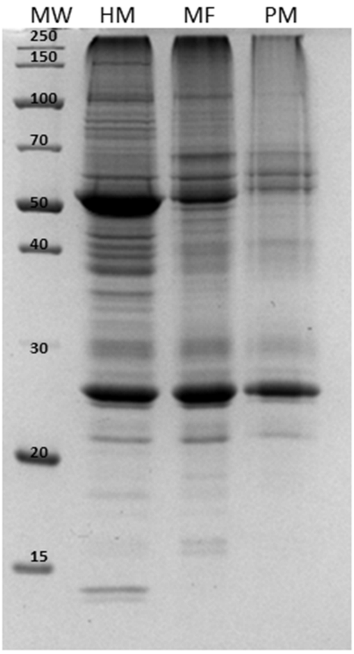

Figure S2: Representative 12% 1D-SDS-PAGE subsequent to PM-associated fraction isolation of 24 h ergosterol-treated Arabidopsis leaves. The gel shows the different fractions obtained after each centrifugation step and the decreasing protein content between the homogenate (HM) -, microsomal (MF) - and the plasma membrane (PM-associated) fractions. Equal volumes were loaded for each fraction and electrophoresed at constant 90 V for 3 h.

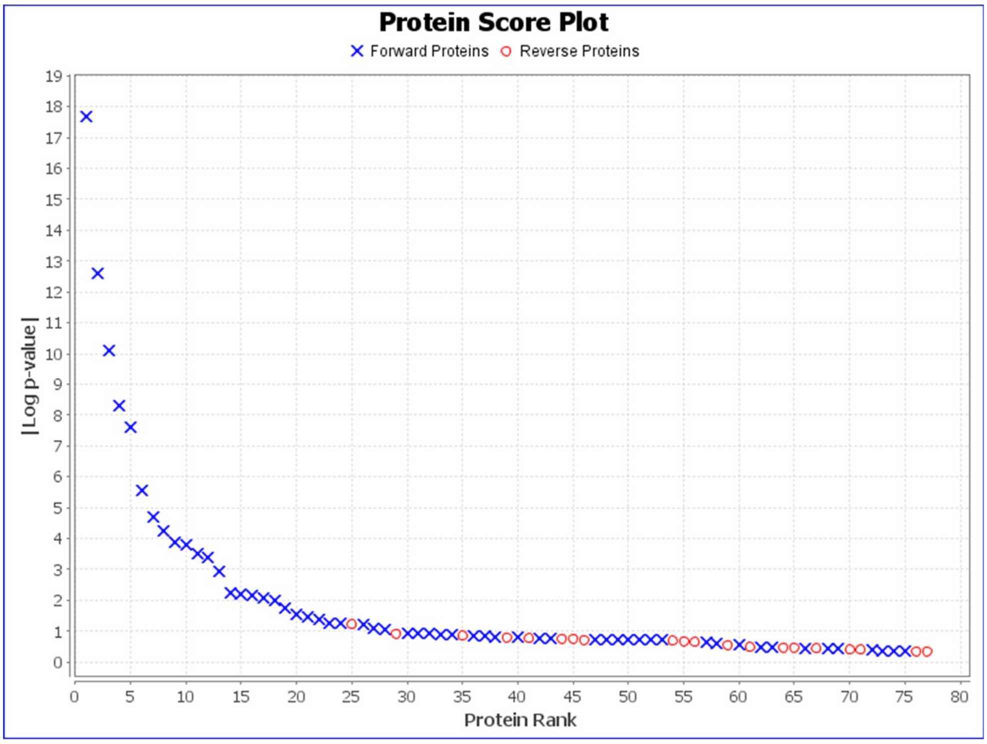

Figure S3: Representation of a protein score plot generated by the Byonic™ software for protein identification. This shows differential abundance of proteins in sample.

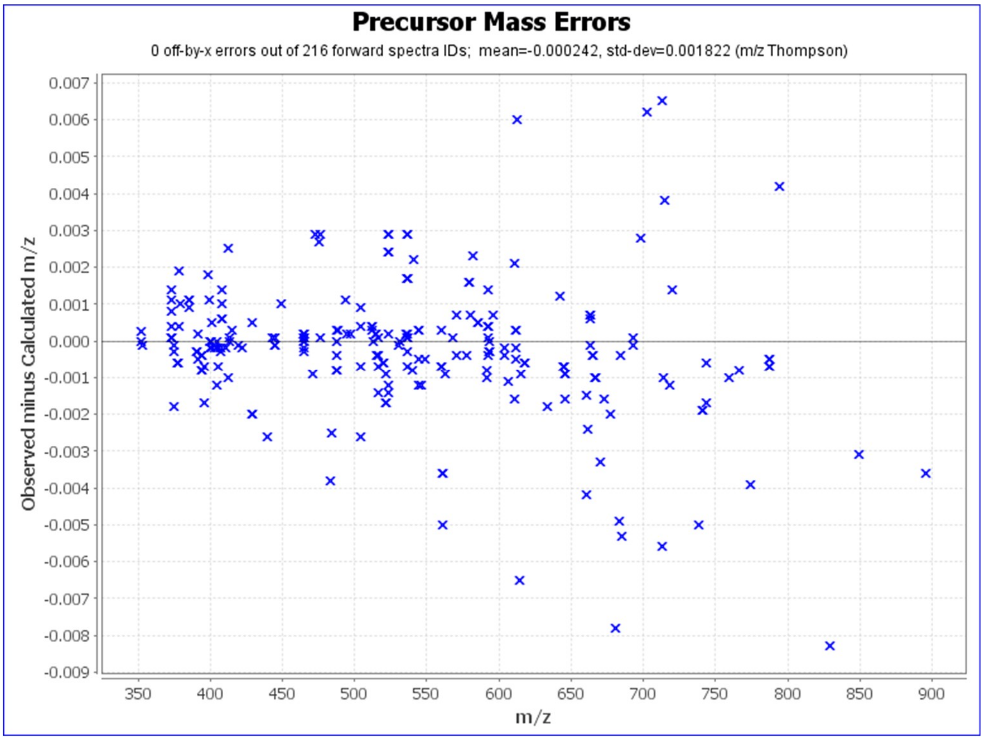

Figure S4: Representation of a mass error loadings plot generated by the Byonic™ software for protein identification. This shows the difference between the calculated mass and the observed mass of the peptides.

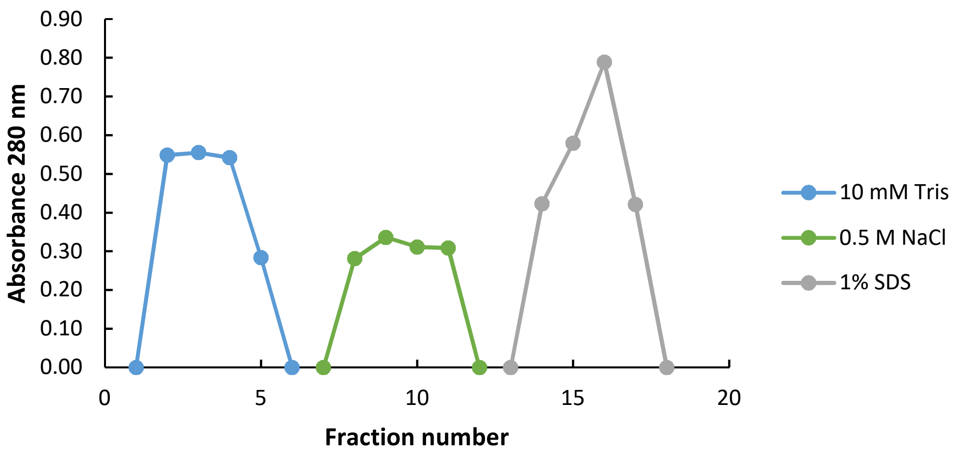

Figure S5: Elution profile of binding events between ergosterol-immobilized MagResyn™ magnetic microspheres and *A. thaliana* PM-associated M proteins for the control. The blue curve represents the absorbance of the flow-through (unbound) fractions eluted with 10 mM Tris-HCl, pH 7.5. The green curve is the absorbance of the weakly bound proteins removed with 0.5 M NaCl and the grey curve represents absorbance of proteins desorbed from the column with 1% SDS solution.

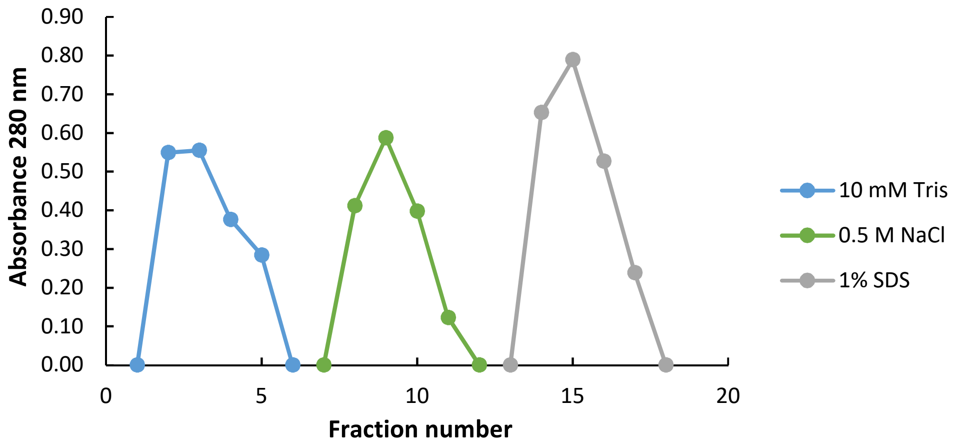

Figure S6: Elution profile of binding events between ergosterol-immobilized MagResyn™ magnetic microspheres and *A. thaliana* PM-associated proteins for the 0 h time point. The blue curve represents the absorbance of the flow-through (unbound) fractions eluted with 10 mM Tris-HCl, pH 7.5. The green curve is the absorbance of the weakly bound proteins removed with 0.5 M NaCl and the grey curve represents absorbance of proteins desorbed from the column with 1% SDS solution

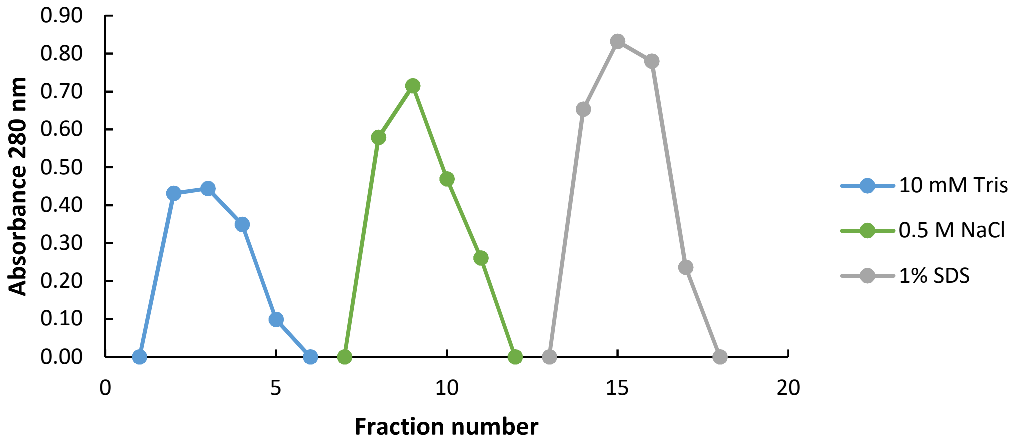

Figure S7: Elution profile of binding events between ergosterol-immobilized MagResyn™ magnetic microspheres and *A. thaliana* PM-associated proteins for the 12 h time point. The blue curve represents the absorbance of the flow-through (unbound) fractions eluted with 10 mM Tris-HCl, pH 7.5. The green curve is the absorbance of the weakly bound proteins removed with 0.5 M NaCl and the grey curve represents absorbance of proteins desorbed from the column with 1% SDS solution.

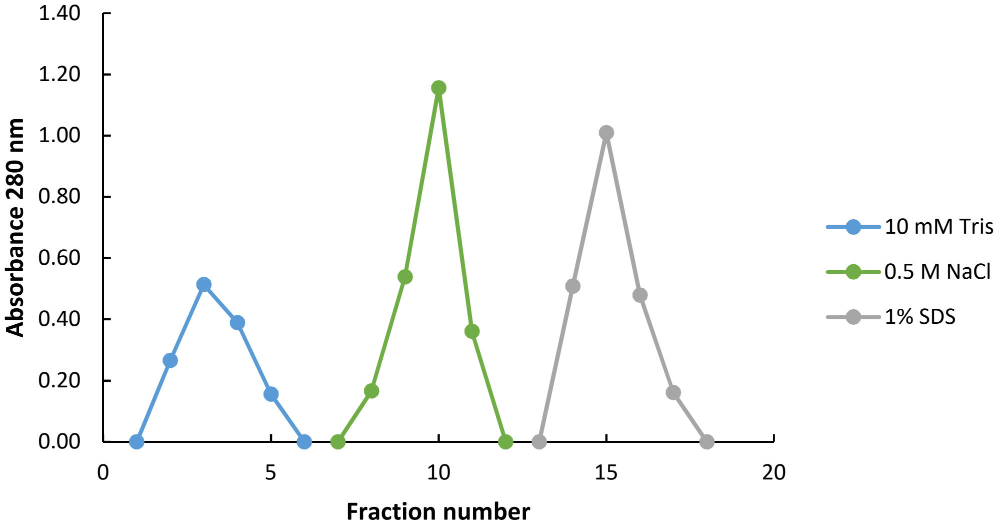

Figure S8: Elution profile of binding events between ergosterol-immobilized MagResyn™ magnetic microspheres and *A. thaliana* PM-associated proteins for the 24 h time point. The blue curve represents the absorbance of the flow-through (unbound) fractions eluted with 10 mM Tris-HCl, pH 7.5. The green curve is the absorbance of the weakly bound proteins removed with 0.5 M NaCl and the grey curve represents absorbance of proteins desorbed from the column with 1% SDS solution.

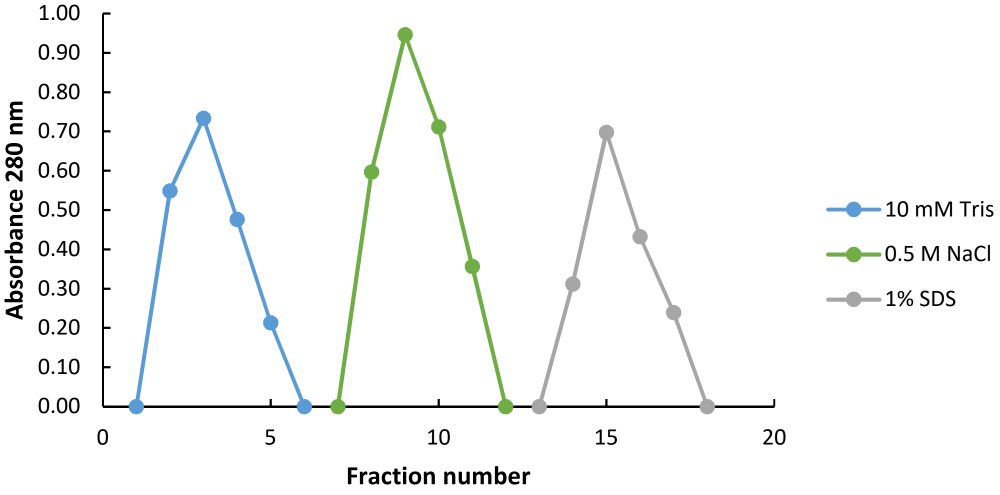

Figure S9: Elution profile of binding events between MagResyn™ magnetic microspheres and *A. thaliana* PM-associated proteins for the negative control (no ergosterol immobilization). The blue curve represents the absorbance of the flow-through (unbound) fractions eluted with 10 mM Tris-HCl, pH 7.5. The green curve is the absorbance of the weakly bound proteins removed with 0.5 M NaCl and the grey curve represents absorbance of proteins desorbed from the column with 1% SDS solution.

Table S1: LC-MS/MS identification of low score *Arabidopsis thaliana* PM-associated candidate proteins interacting with ergosterol immobilized on epoxide magnetic microspheres for control, 0-, 6-, 12- and 24 h samples subsequent to treatment.

Sample no.	Protein	Accession no.	Calculated mass[a] (M+H)	Mass error[b] (ppm)	Byonic™ score[c]	ǀLog probǀ[d]
**Signaling**						
A13	Leucine-rich repeat receptor-like protein kinase PXC1 At2g36570	Q9SJQ1	736.424	0.7	292.90	0.72
A14	Probable LRR receptor-like serine/threonine-protein kinase At1g29720	Q9ASQ6	787.492	-0.2	290.10	0.67
A4	Receptor like protein 46 At4g04220	F4JGB6	1173.647	-1.1	268.3	0.55
A6	Leucine-rich repeat receptor-like serine/threonine/tyrosine-protein kinase SOBIR1 At2g31880	Q9SKB2	1005.573	-0.9	259.5	1.20
A14	Leucine-rich repeat receptor-like protein kinase At2g01210	Q9ZU46	870.541	-2.1	225.50	0.35
A3	Protein BRASSINOSTEROID INSENSITIVE 1 At4g39400	O22476	1043.548	-0.4	220.2	0.40
A10	Receptor-like protein kinase FERONIA At3g51550	Q9SCZ4	530.305	-0.3	188.2	0.77
A3, A8	Plasma membrane-associated cation-binding protein 1 At4g20260	Q96262	536.308	-1.0	178.1	0.48
A10	Tetraspanin-3 At3g45600	Q9M1E7	1186.533	-1.0	179.6	0.72
A12	14-3-3-like protein GF14 omega At1g78300	Q01525	907.525	-0.4	164.9	0.56
A9	Cysteine-rich receptor-like protein kinase 41 At4g00970	O23081	973.531	0.9	86.0	0.61
**Membrane trafficking and transport**						
A1	Ras-related protein RABG3f At3g18820	Q9LS94	1187.621	-0.1	297.6	6.66
A13	ABC transporter C family member 8 At3g21250	Q8LGU1	472.349	-1.0	283.4	0.73
A11	Syntaxin-22 At5g46860	P93654	820.456	-0.7	276.4	0.40
A10	Syntaxin-132 At5g08080	Q8VZU2	805.420	0.6	246.8	0.75
A4	Aluminum-activated malate transporter 5 At1g68600	Q93Z29	430.302	-0.5	246.5	0.98
A2	Copper ion transmembrane transporter At2g37920	Q8LG21	773.513	-0.8	241.6	0.47
A2	PRA1 family protein B4 At2g38360	O80915	1306.700	-1.1	222.0	2.42
A10	Sugar transporter ERD6-like 6 At1g75220	Q9FRL3	777.462	-0.3	221.4	0.83
A14	Patellin-2 At1g22530	Q56ZI2	1078.589	-0.9	198.50	0.37
A3, A4	Auxin transport protein BIG At3g02260	Q9SRU2	731.405	-1.4	196.1	0.16
A7	Putative ABC transporter B family member 8 At3g30875	Q9LHK4	502.324	-0.4	192.9	0.49
A5	ABC transporter C family member 2 At2g34660	Q42093	375.235	-1.6	186.3	0.54
A1	ABC transporter A family member 7 At3g47780	Q9STT5	401.287	-0.4	185.4	0.15
**Structure**						
A12	Actin-4 At5g59730	P53494	976.448	-0.5	197.2	0.69
A1, A9, A10	Actin-3 At3g53750	P0CJ47	945.552	1.1	153.6	0.42
A10	Fasciclin-like arabinogalactan protein 9 At1g03870	Q9ZWA8	1238.586	-0.6	143.7	0.67
**Defense**						
A11	Germin-like protein subfamily 3 member 1 At1g72610	P94040	560.304	-0.9	237.5	0.23
A5	Protein BONZAI 1 At5g61900	Q941L3	1060.615	0.8	228.1	0.24
A12	Dehydrin ERD14 At1g76180	P42763	896.488	-0.9	174.5	0.64
A8	Temperature-induced lipocalin-1 At5g58070	Q9FGT8	1110.531	-0.1	168.0	1.97
A10	Jacalin-related lectin 22 At2g39310	O80950	1191.648	1.4	139.9	0.21

a= the computed M+H precursor mass for the peptide spectrum matches (PSMs).
b= a calculated mass error (parts per million) after correcting the observed M+H (single charged) precursor mass and the computed M+H precursor mass.
c= Byonic score, primary indicator of PSM correctness. Score of 300 is considered to be a significant hit [35].
d= the log p-value of the PSM, which the value should be ≥ 1 for hit to be significant

Table S2: LC-MS/MS identification of *Arabidopsis thaliana* PM-associated candidate proteins interacting with magnetic microspheres for the negative control (no ergosterol immobilization) subsequent to ergosterol treatment.

Protein name	Accession no.	Calculated mass[a] (M+H)	Mass error[b] (ppm)	Byonic™ score[c]	ǀLog probǀ[d]
Photosystem I reaction center subunit XI At4g12800	Q9SUI4	1527.801	0.9	543.30	9.73
Cytochrome b6-f complex subunit 4 Atcg00730	P56774	1166.653	-0.7	521.40	7.89
Chlorophyll A-B binding protein At1g15820	Q9LMQ2	741.451	-1.6	501.90	7.38
NAD(P)-linked oxidoreductase-like protein At1g14345	Q949S6	1232.648	-0.6	451.30	8.20
Photosystem II 22 kDa protein At1g44575	Q9XF91	1123.578	-1.2	385.10	7.16
Protein translocase subunit SECA1 At4g01800	Q9SYI0	1059.543	-0.2	345.90	7.40
UPF0603 protein At1g54780 At1g54780	Q9ZVL6	1057.662	0.8	327.20	6.29
Photosystem I reaction center subunit III At1g31330	Q9SHE8	1225.715	-1.2	331.00	8.35
Chlorophyll a-b binding protein CP29.1 At5g01530	Q07473	1061.522	-0.5	330.00	5.60
Photosystem I reaction center subunit psaK At1g30380	Q9SUI5	932.495	-0.9	329.50	7.35
Photosystem II D2 protein Atcg00270	P56761	1041.605	0.4	320.90	6.70
Protein ACCLIMATION OF PHOTOSYNTHESIS TO ENVIRONMENT At5g38660	Q2HIR7	918.453	-0.2	317.60	7.00
Phytosulfokine receptor 1 At2g02220	Q9ZVR7	1169.664	0.3	283.10	2.25
Acetyl-CoA carboxylase 1 At1g36160	Q38970	401.287	-0.3	172.70	3.42

a= the computed M+H precursor mass for the peptide spectrum matches (PSMs).
b= a calculated mass error (parts per million) after correcting the observed M+H (single charged) precursor mass and the computed M+H precursor mass.
c= Byonic score, primary indicator of PSM correctness. Score of 300 is considered to be a significant hit [35]
d= the log p-value of the PSM, which the value should be ≥ 1 for hit to be significant.

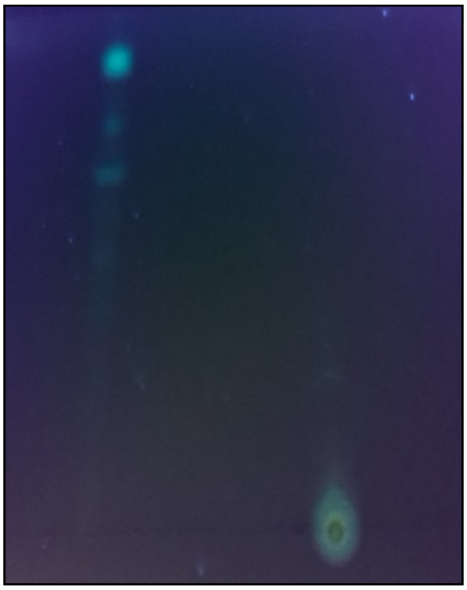

Erg Erg*

Figure S10: Representative thin-layer chromatogram (TLC) of ergosterol and ergosterol-hemisuccinate analyzed with HPTLC. The derivatization of ergosterol was confirmed by comparing 30 mg/mL ergosterol (Erg) and 30 mg/mL ergosterol-hemisuccinate (Erg*), both dissolved in toluene:acetone (70:30, v/v). One µL of each solution was spotted on the plate and the mobile phase was toluene:acetone (70:30, v/v). The plate was visualized under UV at 254 nm.

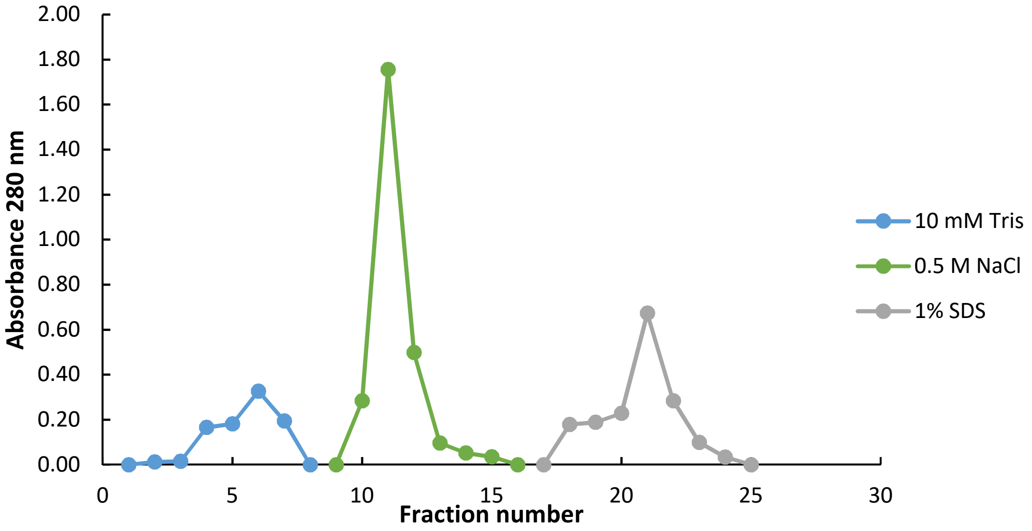

Figure S11: Elution profile of binding events between ergosterol-hemisuccinate immobilized on EAH Sepharose 4B resin and *A. thaliana* PM-associated proteins for the control. The blue curve represents the flow-through (unbound) fractions eluted with 10 mM Tris-HCl, pH 7.5 buffer. The green curve represents the non-specifically bound fractions removed with 0.5 M NaCl in buffer and the grey curve represents the proteins of interest eluted with 1% SDS in buffer.

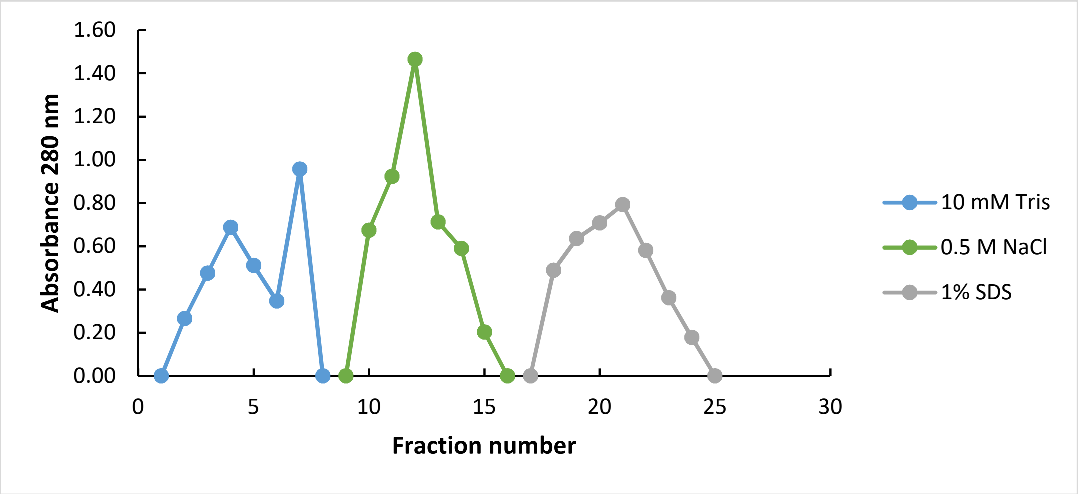

Figure S12: Elution profile of binding events between ergosterol-hemisuccinate immobilized on EAH Sepharose 4B resin and *A. thaliana* PM-associated proteins for the 0 h time point. The blue curve represents the flow-through (unbound) fractions eluted with 10 mM Tris-HCl, pH 7.5 buffer. The green curve represents the non-specifically bound fractions removed with 0.5 M NaCl in buffer and the grey curve represents the proteins of interest eluted with 1% SDS in buffer.

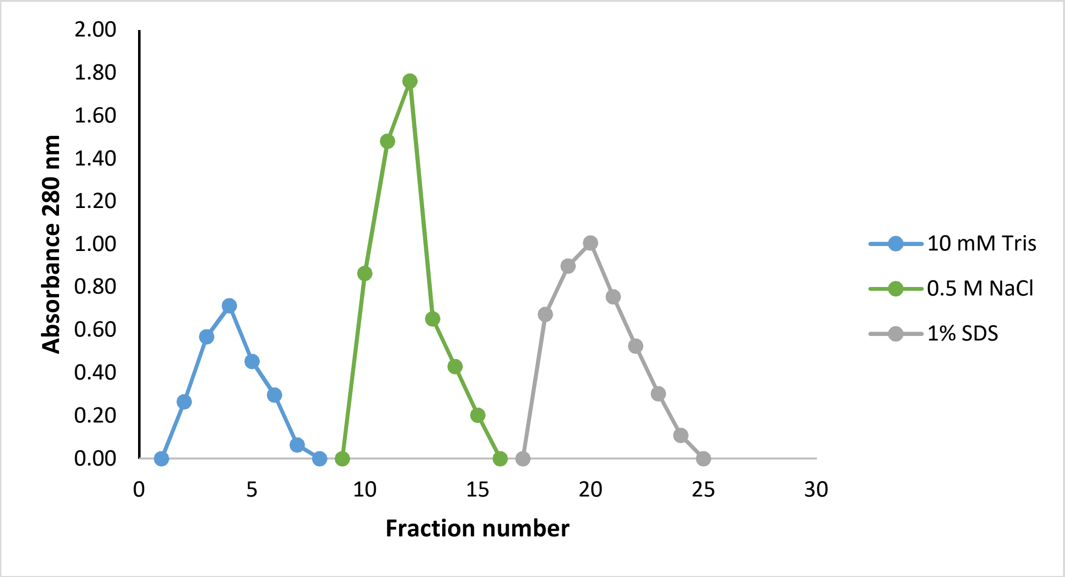

Figure S13: Elution profile of binding events between ergosterol-hemisuccinate immobilized on EAH Sepharose 4B resin and *A. thaliana* PM-associated proteins for the 12 h time point. The blue curve represents the flow-through (unbound) fractions eluted with 10 mM Tris-HCl, pH 7.5 buffer. The green curve represents the non-specifically bound fractions removed with 0.5 M NaCl in buffer and the grey curve represents the proteins of interest eluted with 1% SDS in buffer.

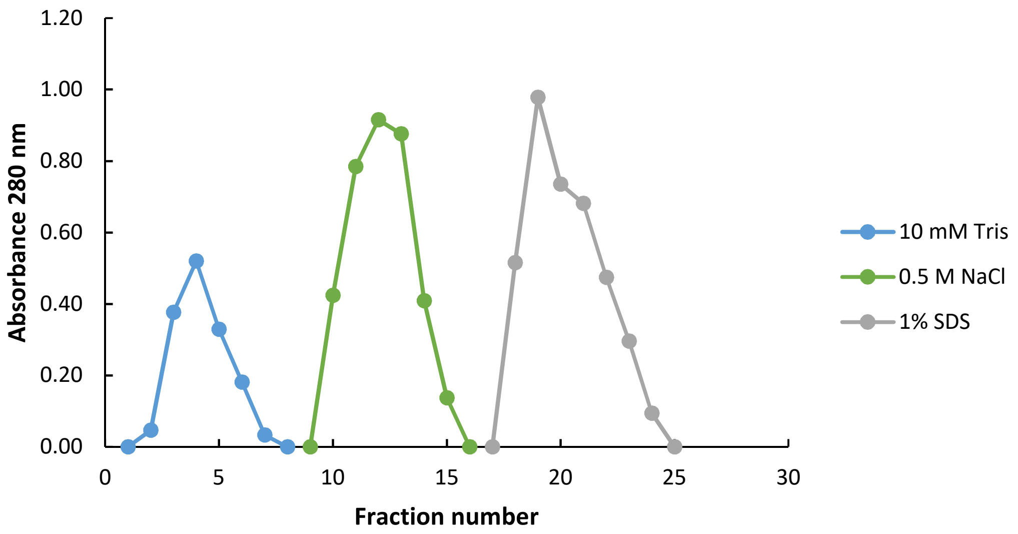

Figure S14: Elution profile of binding events between ergosterol-hemisuccinate immobilized on EAH Sepharose 4B resin and *A. thaliana* PM-associated proteins for the 24 h time point. The blue curve represents the flow through fractions removed with 10 mM Tris-HCl, pH 7.5 buffer. The green curve represents the non-specifically bound fractions removed with 0.5 M NaCl in buffer and the grey curve represents the protein(s) of interest eluted with 1% SDS in buffer.

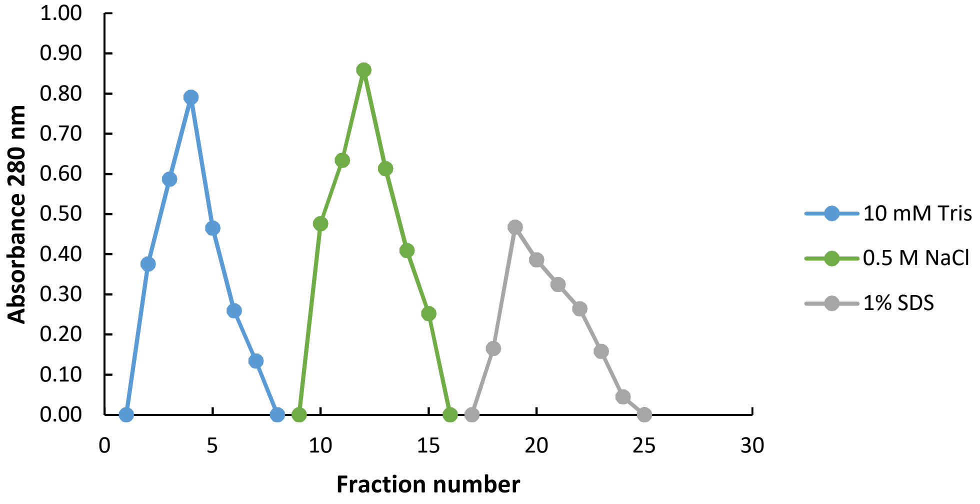

Figure S15: Elution profile of binding events between EAH Sepharose 4B resin and *A. thaliana* PM-associated proteins for the negative control (no ergosterol immobilization). The blue curve represents the flow-through (unbound) fractions eluted with 10 mM Tris-HCl, pH 7.5 buffer. The green curve represents the non-specifically bound fractions removed with 0.5 M NaCl in buffer and the grey curve represents the proteins of interest eluted with 1% SDS in buffer.

Table S3: LC-MS/MS identification of low score *Arabidopsis thaliana* PM-associated candidate proteins, interacting with ergosterol-hemisuccinate immobilized on EAH Sepharose 4B resin for control, 0-, 6-, 12- and 24 h samples subsequent to treatment.

Sample no.	Protein name	Accession no.	Calculated mass[a] (M+H)	Mass error[b] (ppm)	Byonic™ score[c]	∣Log prob∣[d]
**Signaling**						
A6	MAP3K epsilon protein kinase 2 At3g07980	Q9SFB6	1187.699	-0.3	268.00	0.33
A5	Serine/threonine-protein kinase ATM At3g48190	Q9M3G7	418.230	-0.2	191.00	0.36
A1	Putative GTP-binding protein ara-3 At5g59840	Q9FJF1	1316.659	-2.4	158.4	0.46
A6	LRR receptor-like serine/threonine-protein kinase GSO2 At5g44700	Q9FIZ3	379.209	-0.5	118.00	0.44
**Membrane trafficking and transport**						
A4	Auxin transport protein BIG At3g02260	Q9SRU2	409.219	-0.4	308.30	0.78
A2	ABC transporter B family member 15 At3g28345	Q9LHD1	386.240	-0.3	72.40	0.33
A3, A5, A9	ABC transporter B family member 19 At3g28860	Q9LJX0	487.324	-0.2	196.90	0.33
A9	Potassium channel AKT6 At2g25600	Q8GXE6	729.462	-0.1	279.30	0.29
A10	Phospholipid-transporting ATPase 1 At5g04930	P98204	501.340	0.3	273.80	0.34
A7	ABC transporter G family member 41 At4g15215	Q7PC83	401.287	-0.8	197.80	0.54
A5	Protein NRT1/ PTR FAMILY 6.3 At1g12110	Q05085	515.330	-0.7	164.10	0.42
A4	Putative ABC transporter B family member 8 At3g30875	Q9LHK4	502.324	-0.2	150.00	0.86
A5	Auxin transport protein BIG At3g02260	Q9SRU2	373.208	-0.5	70.10	0.38
**Defense**						
A6	Disease resistance protein At4g27190	Q9T048	635.304	1.8	340.80	0.35
**Structure**						
A10	Actin-3 At3g53750	P0CJ47	945.552	0.3	159.5	0.58

a= the computed M+H precursor mass for the peptide spectrum matches (PSMs).
b= a calculated mass error (parts per million) after correcting the observed M+H (single charged) precursor mass and the computed M+H precursor mass.
c= Byonic score, primary indicator of PSM correctness. Score of 300 is considered to be a significant hit [35].
d= the log p-value of the PSM, which the value should be ≥ 1 for hit to be significant.

Table S4: LC-MS/MS identification of *Arabidopsis thaliana* PM-associated candidate proteins interacting with the EAH Sepharose 4B resin for the negative control (no ergosterol immobilization) subsequent to ergosterol treatment.

Protein name	Accession no.	Calculated mass[a] (M+H)	Mass error[b] (ppm)	Byonic™ score[c]	∣Log prob∣[d]
Chlorophyll a-b binding protein 3 At1g29910	Q8VZ87	1265.555	-0.1	590.70	9.62
Photosystem I chlorophyll a/b-binding protein 3-1 At1g61520	Q9SY97	1629.903	-1.3	491.90	9.13
Cytochrome b6-f complex subunit 4 Atcg00730	P56774	1166.653	-0.8	438.10	8.30
Photosystem I reaction center subunit III At1g31330	Q9SHE8	1080.594	0.5	435.50	9.10
NAD(P)-linked oxidoreductase-like protein At1g14345	Q949S6	1232.648	-1.0	413.60	9.43
Photosystem I reaction center subunit XI At4g12800	Q9SUI4	883.536	-0.4	412.00	8.87
TIR-NBS-LRR class disease resistance protein At5g45240	F4KD49	573.361	-0.5	406.10	1.37
Cytochrome b559 subunit alpha Atcg00580	P56779	954.573	0.1	395.10	8.36
Photosystem II protein D1 Atcg00020	P83755	963.453	1.0	365.80	6.71

Nucleoside diphosphate kinase III At4g11010	O49203	529.371	-1.6	361.30	0.67
Rhodanese-like domain-containing protein 9 At2g42220	O48529	900.551	0.4	350.60	7.91
Probable plastid-lipid-associated protein 4 At3g26070	Q9LU85	1130.544	-1.7	341.20	8.00
At3g27700	Q9XF87	3555.753	-2.2	334.20	8.17
Ribulose bisphosphate carboxylase small chain 3B At5g38410	P10798	935.495	1.6	316.60	1.79
Glutamyl-tRNA reductase 2 At1g09940	P49294	1130.642	-1.0	305.20	7.06
C2 and GRAM domain-containing protein At1g03370	Q9ZVT9	515.330	0.1	305.00	0.75
40S ribosomal protein S9-1 At5g15200	Q9LXG1	1047.583	-0.1	304.80	2.62

a= the computed M+H precursor mass for the peptide spectrum matches (PSMs).

b= a calculated mass error (parts per million) after correcting the observed M+H (single charged) precursor mass and the computed M+H precursor mass.

c= Byonic score, primary indicator of PSM correctness. Score of 300 is considered to be a significant hit [35].

d= the log p-value of the PSM, which the value should be ≥ 1 for hit to be significant.
